# Integrated machine learning and single-cell analysis reveal the prognostic and therapeutic potential of SUMOylation-related genes in ovarian cancer

**DOI:** 10.3389/fimmu.2025.1577781

**Published:** 2025-06-04

**Authors:** Zhengrong Deng, Yicong Xu, Peidong Zhang, Yixiang Peng, Jiaxing Tan, Zihang Chen, Yimei Ma

**Affiliations:** ^1^ Key Laboratory of Birth Defects and Related Diseases of Women and Children (Sichuan University) Ministry of Education, West China Second University Hospital of Sichuan University, Chengdu, China; ^2^ Department of Obstetrics and Gynecology, West China Second University Hospital of Sichuan University, Chengdu, China; ^3^ Department of Anesthesiology, West China Second University Hospital of Sichuan University, Chengdu, China; ^4^ State Key Laboratory of Biotherapy and Cancer Center, West China Hospital, Sichuan University, Chengdu, China; ^5^ Department of Computer Science and Technology, Civil Aviation University of China, Tianjin, China; ^6^ Division of Nephrology, Department of Medicine, West China Hospital, Sichuan University, Chengdu, China; ^7^ Department of Pathology, West China Hospital, Sichuan University, Chengdu, China; ^8^ Department of Hematology and Oncology, West China Second University Hospital of Sichuan University, Chengdu, China

**Keywords:** sumoylation, machine learning, single-cell RNA-seq, clinical cohort, chemoresistance

## Abstract

**Introduction:**

Ovarian cancer (OC) exhibits high mortality and chemoresistance rates, underscoring the urgent need for precise prognostic biomarkers and novel therapeutic targets. SUMOylation, crucial in cellular stress responses, is frequently dysregulated in various cancers. This study aims to characterize SUMOylation and its regulators in OC and identify potential biomarkers and therapeutic targets.

**Methods:**

In this study, using multi-omics data, we characterized the unique features of SUMOylation in OC and revealed the association between SUMOylation-related genes (SRGs) and OC malignancy. We conducted integrated machine learning and single-cell RNA sequencing data analysis to identify key SRGs and explored their functional characteristics. The prognostic potential of these SRGs was confirmed in ID8 mouse models and in samples from 213 OC patients at West China Second Hospital.

**Results:**

An integrated machine learning framework identified 22 prognostic-related SRGs from the TCGA-OV cohort. Further single-cell analysis refined these findings, pinpointing five SRGs as biomarkers closely associated with OC cell function, metabolism and the tumor microenvironment. In cancer cells, the expression of four SRGs (*PI3, AUP1, CD200* and *GNAS*) is closely associated with epigenetic regulation and epithelial-mesenchymal signaling. Notably, we found that *AUP1* overexpression may contribute to chemoresistance in OC. In the tumor microenvironment, CD8^+^ cytotoxic T cell with high *CCDC80* (another SRG) expression exhibit inhibited cytotoxicity activity.

**Discussion:**

Overall, five SRGs were identified and further evaluated as potential prognostic and therapeutic targets, offering deeper insights into precision oncology for OC.

## 1 Introduction

Ovarian cancer (OC) is a leading cause of gynecologic cancer-related mortality (1), with a poor prognosis and a 5-year survival rate of approximately 20% in advanced stages (2). Chemoresistance to standard platinum-based chemotherapy further contributes to poor survival, particularly in advanced cases (3, 4). Despite significant advances in targeted therapies, such as the combination of bevacizumab with carboplatin and paclitaxel, which has improved survival in stage IV patients (5), the high heterogeneity of ovarian cancer limits the overall response (6). Moreover, immunotherapy has shown only modest effectiveness in ovarian cancer and lacks FDA approval (7). Thus, studies on novel prognostic signatures and molecular biomarkers, with a focus on new molecular mechanisms, are urgently needed.

One such mechanism is posttranslational protein modification by small ubiquitin-like modifier (SUMO), termed SUMOylation, which plays a crucial role in cellular responses to stress and is altered in many cancers (8, 9). Inhibiting SUMOylation activity in acute myeloid leukemia can resensitize cancer cells to genotoxic chemotherapy (10). However, another study indicated that high levels of SUMOylation repress the effects of TGFβ signaling on E-cadherin expression, revealing a negative regulatory mechanism for epithelial–mesenchymal transition (EMT), thus inhibiting cancer progression and metastasis (11). Therefore, SUMOylation plays complex regulatory roles in protumorigenic signaling, gene regulatory networks, antitumor immunity and inflammatory cytokine production, exerting both agonistic and antagonistic effects depending on the pathway (8, 12). However, research investigating SUMOylation in ovarian cancer remains scarce.

Machine learning, a branch of artificial intelligence (AI) focused on predicting data patterns via algorithms, has long played a crucial role in cancer phenotyping, therapy and advanced techniques such as signature extraction and prognosis prediction (13). Many studies have used machine learning algorithms and TCGA data to pinpoint therapeutic targets to improve ovarian cancer prognosis (14). However, methods based on single-model pattern recognition fail to meet the growing demand for precision medicine, as they often lack in-depth consideration of target mechanisms and sufficient clinical validation, thereby limiting their clinical translation (15). Conversely, single-cell RNA sequencing has emerged as a powerful tool for delineating distinct functional states at the cellular level in ovarian cancer (16). Integrating multiple machine learning approaches with single-cell data holds promise for identifying more precise therapeutic targets and revealing intricate gene regulatory networks in ovarian cancer.

In this study, via multi-omics data, we revealed unique characteristic of SUMOylation in ovarian cancer and explored the potential association between SUMOylation-related genes and ovarian cancer malignancy. Through the application of integrated machine learning techniques and single-cell analysis, we further identified SRGs closely associated ovarian cancer cell function and the tumor immune microenvironment (TIME). Additionally, we identified a biomarker that may indicate poor postoperative chemotherapy prognosis. This work holds promise for improving therapeutic outcomes, enhancing patient prognosis and advancing precision medicine in oncology.

## 2 Materials and methods

### 2.1 Data collection and preprocessing

Publicly available gene expression profiles and complete clinical annotations for the TCGA-OV cohort were obtained from The Cancer Genome Atlas (TCGA) database (https://portal.gdc.cancer.gov/) using the TCGAbiolinks R package (version 2.34.0). Given that the TCGA-OV gene expression data are derived from high-throughput RNA sequencing (RNA-seq), we utilized fragments per kilobase of exon model per million mapped fragments (FPKM) values, as they are more comparable to microarray-derived expression data and facilitate cross-sample normalization. For microarray datasets, expression matrices and corresponding clinical data were retrieved from the Gene Expression Omnibus (GEO; https://www.ncbi.nlm.nih.gov/geo/) using the GEOquery package (version 2.70.0), specifically for the datasets GSE13876, GSE17260, GSE19829, and GSE26712. Gene annotation and probe ID conversion for these microarray platforms were performed using the tinyarray package (version 2.3.1). Additionally, single-cell RNA sequencing (scRNA-seq) data from ten patients with advanced ovarian cancer were obtained from the GSA-Human database (https://ngdc.cncb.ac.cn/gsa-human/) under the accession number PRJCA005422 and were reanalyzed in the present study. All the code generated for analysis is available through Zenodo, DOI: 10.5281/zenodo.13152228 (https://zenodo.org/records/13152229).

### 2.2 Analysis of scRNA-seq data

Single-cell RNA sequencing (scRNA-seq) data were processed and analyzed using the Seurat R package (version 4.4.0). Raw data were first converted into Seurat objects, followed by quality control to remove low-quality nuclei. Specifically, cells were excluded if they had fewer than 500 or more than 3,000 detected genes, or if more than 20% of total transcripts originated from mitochondrial or ribosomal genes. Data normalization was conducted using the NormalizeData function with default parameters. The 2,000 most variable genes were identified for principal component analysis (PCA), which was used for dimensionality reduction. To address batch effects across samples, the datasets were integrated using the FindIntegrationAnchors and IntegrateData functions. After integration, clustering analysis was conducted using the FindNeighbors and FindClusters functions. The resolution parameter was adjusted to optimize cluster granularity, enabling the identification of distinct cell populations. Clusters were visualized using uniform manifold approximation and projection (UMAP) based on the top principal components. Cell types were annotated according to canonical marker genes and supported by reference to previous studies. Differential gene expression between clusters or conditions was assessed using the FindMarkers function, applying the Wilcoxon rank-sum test. Genes with an absolute log_2_(fold change) greater than 0.1 and an adjusted p-value less than 0.05 were considered significantly differentially expressed.

### 2.3 Pathway analysis

Gene Ontology (GO) and Kyoto Encyclopedia of Genes and Genomes (KEGG) annotations were retrieved using the “org.Hs.eg.db” R package (version 3.17). Differentially expressed genes were subjected to functional enrichment analysis using the “clusterProfiler” package (version 3.14.3). Gene sets representing functional pathways were obtained from the Molecular Signatures Database (MSigDB). To assess pathway activity at the bulk RNA-seq level, Gene Set Variation Analysis (GSVA) was performed using the GSVA package (version 1.46.0). For single-cell RNA-seq data, pathway activity was evaluated using the AUCell package (version 3.16), with the parameter aucMaxRank set to 5% to determine the top-ranking genes contributing to each pathway activity score.

### 2.4 Identification of malignant epithelium and pseudotime trajectory analysis

Copy number variations (CNVs) were inferred using the CopyKAT R package, with normal epithelial cells designated as the reference population. CopyKAT was executed with default parameters to distinguish malignant from non-malignant epithelial cells based on large-scale chromosomal aneuploidy. The resulting copy number alterations (CNAs) were extracted and visualized as heatmaps using the pheatmap R package, in which genes were ordered according to chromosomal location and cells were grouped by cell type to enhance interpretability. Pseudotime trajectory analysis was conducted via the Monocle2 package (version 2.28.0). SUMOylation-related genes are temporal markers of differential expression.

### 2.5 Construction of integrated machine learning model

To identify prognostic SUMOylation-related genes (SRGs), univariate Cox regression analysis was first performed using the “survival” R package (version 3.5.7), with statistically significant SRGs selected based on a threshold of p < 0.05. Next, to construct a robust prognostic model, 80 algorithmic combinations derived from 10 distinct machine learning algorithms were applied to the selected SRGs within the TCGA-OV cohort. These algorithms included CoxBoost, elastic net (Enet), generalized boosted regression modeling (GBM), least absolute shrinkage and selection operator (Lasso), partial least squares regression for Cox (plsRcox), random survival forest (RSF), Ridge regression, supervised principal components (SuperPC), stepwise Cox regression, and survival support vector machine (survival-SVM). Specific hyperparameters for model training and fitting were as follows: RSF (ntree = 1000, nodesize = 4, splitrule = “logrank”); Enet (α values ranging from 0.1 to 0.9 with 0.1 increments, λ selected via 10-fold cross-validation); Lasso and Ridge (α = 1 and α = 0, respectively, with λ selected through 10-fold cross-validation); Stepwise Cox (three directions tested: forward, backward, and both); CoxBoost (number of boosting steps determined by 10-fold cross-validation); survival-SVM (gamma.mu = 1); GBM (n.trees = 10,000, interaction.depth = 3, shrinkage = 0.001, n.minobsinnode = 10); SuperPC (threshold determined by 10-fold cross-validation, s0.perc = 0.5); and plsRcox (number of components selected via 10-fold cross-validation).

To evaluate model performance, the average concordance index (C-index) across the training, test, and validation datasets was calculated for each algorithmic combination. The combination yielding the highest average C-index was considered optimal and selected for subsequent model development. Using the SRGs identified through this optimal combination, a multivariate Cox regression analysis was conducted via the “survival” R package to construct the final SUMOylation-related gene model (SRGM), defined by the formula: SRGM score = Σ(C_i_ × E_i_), where C_i_ denotes the regression coefficient of each gene and E_i_ its expression level. The prognostic power of the SRGM was then validated across five independent ovarian cancer cohorts through Kaplan–Meier survival analyses.

In addition, previously published mRNA-based prognostic models for ovarian cancer were retrieved from PubMed up to September 2024. For each of these models, the corresponding risk scores were computed based on their reported formulae. Kaplan–Meier analysis was subsequently used to assess their prognostic significance across the training and validation cohorts. Finally, the C-index values of these models were compared with those of our SRGM in each cohort to comprehensively evaluate its predictive advantage.

### 2.6 NicheNet analysis

For NicheNet analysis, the normalized gene expression matrix was used as input. Ligand activity was predicted using the predict_ligand_activities function with default parameters, enabling the identification of potential ligand–receptor interactions and their downstream target genes. This analysis facilitated the inference of key signaling interactions between sender and receiver cell populations within the tumor microenvironment.

### 2.7 Immune microenvironment analysis and prediction of immunotherapy response

To evaluate immune infiltration between high- and low-SRGM score groups, single-sample Gene Set Enrichment Analysis (ssGSEA) was performed using predefined immune cell-type-specific gene signatures, as listed in **Supplementary Table S9**. The Tumor Immune Dysfunction and Exclusion (TIDE, http://tide.dfci.harvard.edu/) algorithm was applied to predict patient responses to immune checkpoint inhibitor (ICI) therapy based on the gene expression count matrix of the TCGA-OV cohort. A higher TIDE score was indicative of a better predicted response to immunotherapy. A score of 0 was used as the threshold to distinguish between predicted responders and non-responders.

### 2.8 Cell culture and transfection

The murine ovarian cancer cell line ID8 was cultured in Dulbecco’s modified Eagle medium (DMEM, Gibco) supplemented with 10% fetal bovine serum (FBS), 100 U/mL penicillin-streptomycin, and 2 mM L-glutamine. Primary ovarian cancer cells were isolated from tumor lesions following previously published protocols (17). For lentiviral transduction, viral particles were obtained from HANBIO (Shanghai, China). The overexpression vector was constructed as pHBLV-CMV-MCS-[Gene of Interest]-EF1-Luc-T2A-Puromycin, and the knockdown vector was based on pHBLV-U6-MCS-[Gene of Interest]-EF1-Luc-T2A-Puromycin. Transductions were performed according to the manufacturer’s instructions, and cells were selected with 4 μg/mL puromycin starting at 72 hours post-infection and maintained under selection until uninfected control cells were eliminated. Expression was confirmed by western blotting prior to further experiments.

### 2.9 Western blotting

Total protein was extracted from cells via RIPA buffer and subsequently separated via sodium dodecyl sulfate–polyacrylamide gel electrophoresis (SDS–PAGE). Following separation, the proteins were transferred to polyvinylidene fluoride (PVDF) membranes that had been preactivated with methanol. The membranes were incubated with blocking buffer at room temperature for 1 hour, followed by overnight incubation at 4°C with primary antibody mixture, which was diluted according to the manufacturer’s instructions. The next day, after washing with a mixture of tris-buffered saline and Tween 20 (TBST) four times for 15 minutes each, the membranes were incubated with an HRP-conjugated secondary antibody solution at room temperature for 50–70 minutes. Detection was performed via the use of an enhanced chemiluminescence (ECL) reagent according to the manufacturer’s protocol. Primary antibodies against the following targets were used: PI3 (Abcam, 1:1000, ab81681), AUP1 (Osenses, 1:100, OSU00005G), CD200 (Abcam, 1:1000, ab254193), GNAS (Abcam, 1:1000, ab204996), SUMO1 (Abcam, 1:1500, ab133352), and SUMO2/3 (Abcam, 1/1000, ab81371). All experiments were independently repeated three times with consistent results (trends).

### 2.10 Real-time quantitative polymerasechain reaction

Total RNA was isolated from frozen tumor tissues using TRIzol reagent (Invitrogen, CA, USA). Based on the RNA’s concentration and integrity, complementary DNA (cDNA) was synthesized using the PrimeScript™ RT Master Mix (#RR036A, Takara, Beijing, China). Quantitative real-time PCR (qPCR) was carried out on the 7500 Real-Time PCR System (Applied Biosystems, CA, USA) employing TB Green^®^ Premix Ex Taq™ (#RR420B, Takara). Primer sequences, designed and synthesized by Sangon Biotech (Shanghai, China), are provided in **Supplementary Table S16**. The relative expression levels of the genes were calculated utilizing the 2^–(ΔΔCT)^ method, with *GAPDH* serving as the internal reference gene for the PCR data.

### 2.11 *In vivo* animal studies

Five-week-old female C57BL/6 mice were purchased from GemPharmatech (Chengdu, China) and maintained under specific pathogen-free (SPF) conditions. After a 10-day acclimatization period, an intraperitoneal injection of 5 × 10^6^ lentivirus-transduced ID8 cells was performed. Mice were randomly assigned to groups. For SUMOylation inhibition experiments, mice received their first tail vein injection of TAK981 (7.5 mg/kg, MedChemExpress) or saline three days prior to tumor implantation, followed by weekly intravenous injections until the endpoint (30 days). For chemotherapy resistance experiments, a chemotherapy regimen of paclitaxel combined with carboplatin (TC) was used, consistent with the clinical cohorts. Starting from day 7 post-tumor implantation, mice received intraperitoneal injections of carboplatin (30 mg/kg, once weekly) and paclitaxel (8 mg/kg, twice weekly) or saline, continuing until the study endpoint. Tumor burden was monitored using the PerkinElmer IVIS Lumina III system.

### 2.12 Patients and clinical samples

The patient screening process is detailed in **Supplementary Figures S6A, B**. All patients were treated at West China Second University Hospital of Sichuan University, and the study was approved by the ethics committee (Approval No. 2024(085)), with informed consent obtained from each patient. The study does not involve clinical trials and did not impose any additional harm or burden on the patients. Formalin-fixed, paraffin-embedded (FFPE) tissue sections for immunohistochemistry were obtained from 60 ovarian cancer patients who underwent surgery between December 2017 and June 2019. Additionally, FFPE samples from 153 patients who underwent surgery between January 2014 and January 2017 were used to construct tissue microarrays and perform immunofluorescence staining. Primary ovarian cancer cells were extracted from fresh tumor samples of chemotherapy-naive patients who underwent surgery between February 2024 and May 2024.

### 2.13 Immunohistochemistry and Immunofluorescence

Tissue sections were deparaffinized with xylene and rehydrated through ethanol gradients. Antigen retrieval was performed in citrate buffer (pH 9.0) using a pressure cooker, followed by blocking with 5% BSA. The sections were incubated overnight at 4°C in a humidified chamber with primary antibody solution with dilution according to the manufacturer’s instructions. For IHC, the following day, sections were stained using DAB staining kit (Absin, Cat# abs9210) after washing. For IF, sections were incubated with a pre-prepared mixture of fluorescent secondary antibodies, then stained with DAPI. Prior to mounting, the sections were incubated with a tissue autofluorescence quenching agent (Servicebio, Cat# G1221). Primary antibodies: PI3 (Abcam, 1:800, ab81681), AUP1 (Osenses, 1:100, OSU00005G), CD200 (Abcam, 1:1000, ab254193), CCDC80 (Abcam, 1:100, ab224050), GNAS (Abcam, 1:1000, ab204996). Secondary antibodies: Goat Anti-Rabbit IgG H&L (HRP) (Abcam, 1:1000, ab6721), Goat Anti-Mouse IgG H&L (HRP) (Abcam, 1:3000, ab205719), Donkey Anti-Goat IgG H&L (HRP) (Abcam, 1:1500, ab6885). Panoramic images were obtained using the Pannoramic MIDI digital slide scanner system. Images were captured at 40× magnification using SlideViewer software and analyzed with ImageJ (version 1.8.0). The process was conducted under the guidance of two experienced pathologists.

### 2.14 Statistical analysis

All data processing, statistical analysis and plotting were conducted in R software (version 4.2.2). Correlations were assessed via Pearson’s correlation coefficients. The chi-square test was applied to compare categorical variables and continuous variables were compared through the Wilcoxon rank-sum test. Cox regression and Kaplan–Meier analyses were performed via the survminer package. The time-dependent area under the ROC curve (AUC) for survival variables was determined via the timeROC package (version 0.4.0). All the statistical tests were two-sided. The threshold for statistical significance was set at p <0.05. *, ** and *** indicate p <0.05, p <0.01 and p <0.001, respectively.

## 3 Results

### 3.1 Unique characteristics of SUMOylation in ovarian cancer

**Figure 1:**
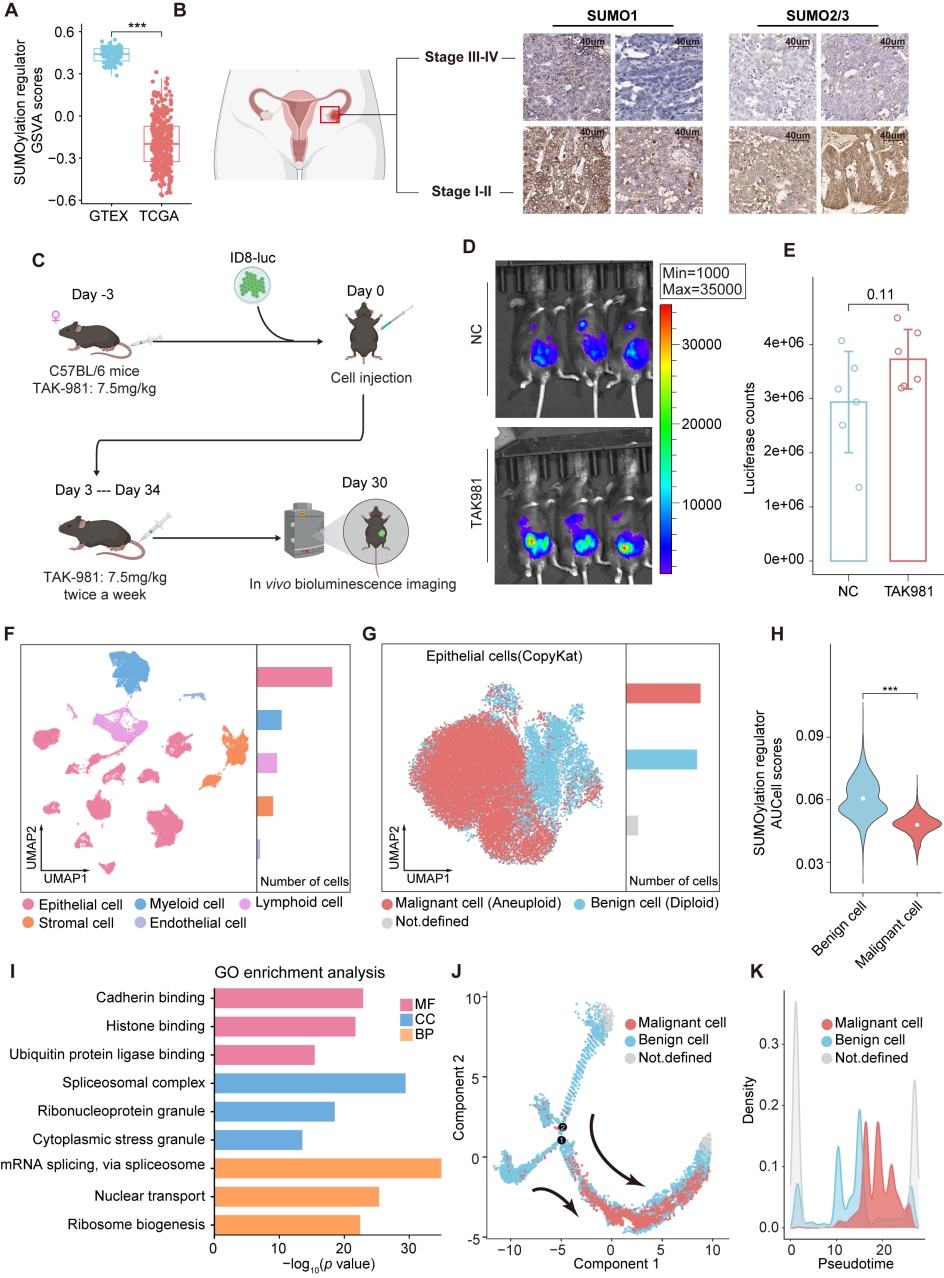
SUMOylation characteristic in ovarian cancer. **(A)** Boxplot comparing the GSVA scores of SUMOylation regulators between ovarian cancer and normal ovarian tissues. **(B)** Immunohistochemical staining of SUMO1 and SUMO2/3 in early and advanced stage ovarian cancer. **(C)** Design of the *in vivo* experiments. **(D)** Bioluminescence images showing the tumor burden of C57BL/6 mice following inoculation with luciferase tagged ID8 cells and subsequent treatment with TAK981 or saline. **(E)** The tumor burden was evaluated by quantification of total flux with Living Image software. **(F)** UMAP plot of 45146 cells from ovarian cancer (primary tumors) grouped into 5 clusters according to cell type. The bar chart on the right displays the relative abundance of each cell type. **(G)** Epithelial cells are reclustered and presented in UMAP plots. The CopyKAT algorithm was utilized to identify aneuploid (malignant) and diploid (benign) cells in ovarian cancer. **(H)** Violin plots representing the differences in the expression of SUMOylation regulators between malignant and benign epithelial cells. **(I)** GO analysis of the SRGs in epithelial cells. **(J)** Monocle trajectory analysis of epithelial cells. The arrow indicates the pseudotime trajectory of epithelial cells from a benign state to a malignant state. **(K)** The density plot reveals changes in the relative abundance of malignant and benign cells across pseudotime. ***, P < 0.001.

To explore the role of SUMOylation in ovarian cancer, we first obtained the gene set of SUMOylation regulators from MSigDB (**Supplementary Table S1**). Gene set variation analysis (GSVA) (18) was conducted to assess the expression levels of SUMOylation regulators in ovarian cancer and normal ovarian samples via the TCGA and GTEx databases (**Figure 1A**), revealing that the expression scores of SUMOylation regulators were relatively lower in ovarian cancer compared to normal ovarian tissues. Since the activity of SUMOylation regulators is closely associated with global SUMOylation levels, we performed IHC for SUMO1 and SUMO2/3 on FFPE tissue sections from ovarian cancer patients at different stages. The results revealed that the global level of SUMOylation was relatively lower in advanced ovarian cancer (**Figure 1B**).

We then employed the widely used ID8 mouse model to evaluate the efficacy of SUMO inhibitors for ovarian cancer (**Figure 1C**), in which a total of 5×10^6^ ID8-luc cells were injected intraperitoneally into immunocompetent C57BL/6 mice. The mice were treated with the SUMOylation inhibitor TAK-981 (7.5 mg/kg) or saline (negative control, NC) starting prior to tumor implantation and continued twice weekly until the study endpoint, as TAK-981 has also been reported to effectively inhibit tumor growth in other cancers (19, 20). However, we found no significant differences at the endpoint and the tumor burden in the SUMOylation inhibition group showed an increasing trend compared to the NC group (**Figures 1D, E**).

To further explore the unique characteristics of SUMOylation in ovarian cancer, we analyzed scRNA-seq data from PRJCA005422 (21), comprising 45,146 cells from primary tumor samples of ten patients with advanced ovarian cancer (**Figure 1F**, **Supplementary Figure S1**). Five distinct cell clusters were identified and annotated according to the expression of classical marker genes (21), the cell types included epithelial cells, myeloid cells, lymphocytes, stromal cells and endothelial cells. We reclustered epithelial cells and distinguished malignant from normal cells based on copy number variations (CNVs) using the CopyKAT algorithm (**Figure 1G**). SUMOylation regulators’ expression was quantified via AUCell. Differential analysis revealed that malignant ovarian cancer cells presented relatively low SUMOylation regulators AUCell scores, which is consistent with our previous findings (**Figures 1H, A**).

We then performed differential expression analysis on epithelial cell groups with high and low expression of SUMOylation regulators and identified 2,538 SUMOylation-related genes (SRGs) (**Supplementary Table S2**). Gene Ontology (GO) enrichment analysis revealed that the SRGs were significantly enriched in pathways involved in protein synthesis and ubiquitin ligase activity regulation, underscoring the broad regulatory effect of SUMOylation on cellular metabolism and functions of ovarian cancer (**Figure 1I**). We analyzed the changes in epithelial cell features during cancer progression by inferring their state trajectories via Monocle, with SRGs used as temporal markers of differential expression. Two primary trajectories from benign to malignant cells were identified across pseudotime (**Figures 1J, K**), underscoring the close association of SRGs with the malignancy of ovarian epithelial cells.

Overall, although numerous studies have revealed the oncogenic effects of elevated SUMOylation in various hematologic (22, 23) and solid tumors (19, 24), SUMOylation inhibition therapy may not achieved the expected outcomes in ovarian cancer. Considering the close association between SRGs and the malignancy of ovarian epithelial cells, identifying potential therapeutic targets and prognostic biomarkers from SRGs may be a promising direction.

### 3.2 Integrated machine learning identified 22 prognostic SRGs

To identify key SRGs, particularly those with consistent roles across diverse cohorts of ovarian cancer patients, we analyzed five datasets comprising patients from different ethnic backgrounds. Genes with absolute z-scores greater than 0.01 across all datasets were intersected with SRGs, resulting in a total of 1,722 genes (**Supplementary Figure S2A**, **Supplementary Table S3**). Using survival data from TCGA-OV, we performed univariate Cox proportional hazards analysis and identified 124 SRGs potentially associated with ovarian cancer patient survival for further investigation (**Supplementary Figure S2B**, **Supplementary Table S4**).

**Figure 2:**
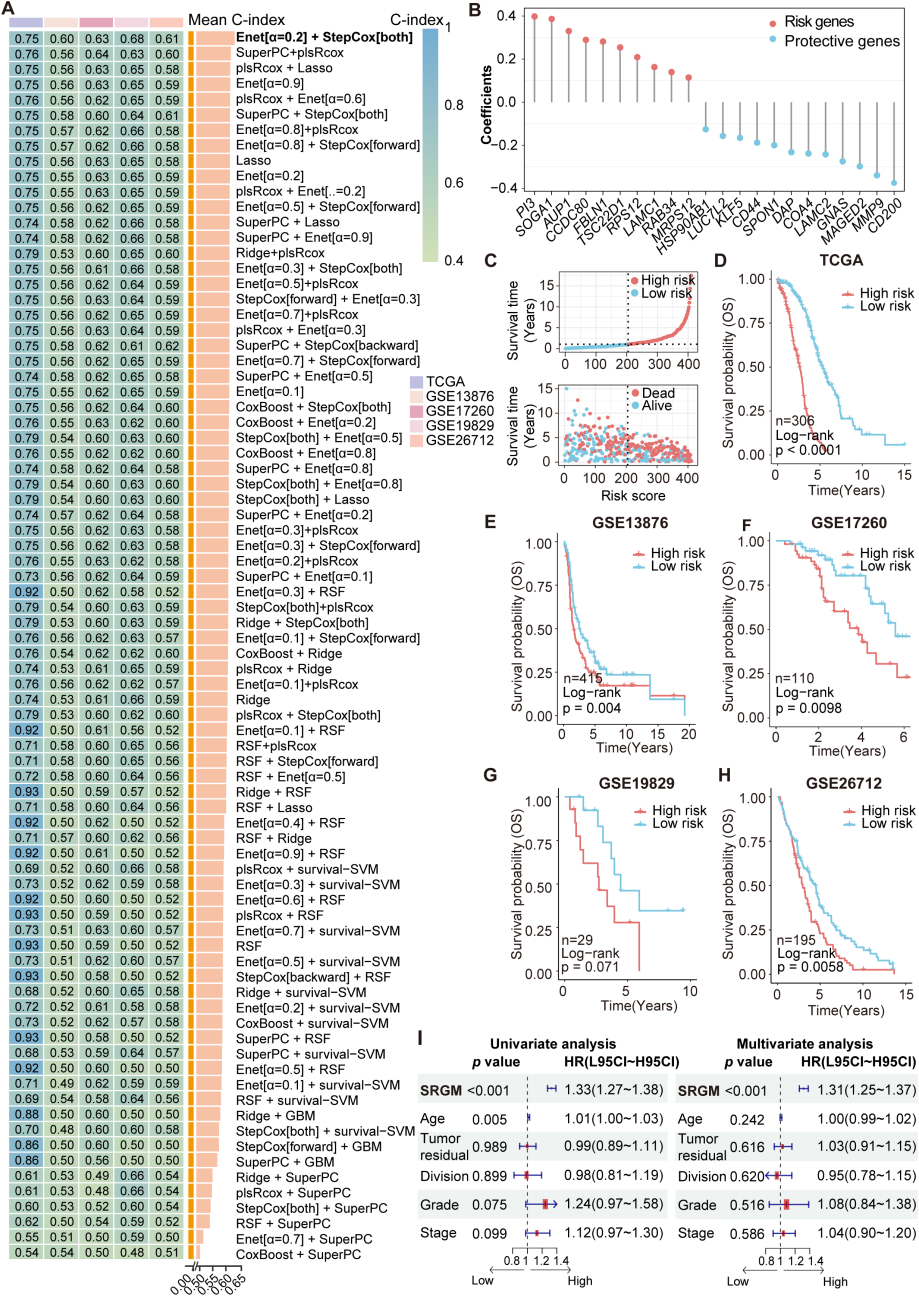
Construction and validation of a machine learning model to identify survival-associated SUMOylation-related genes. **(A)** A total of 80 kinds of prediction models were constructed via the LOOCV framework, and the C-index of each model across all the validation datasets was further calculated. **(B)** Coefficients of 22 SRGs ultimately obtained via Enet (α=0.2) and stepwise Cox regression (both). **(C)** Risk factor plot displaying the association between the SRGM score and survival. **(D–H)** Kaplan–Meier curves of overall survival according to the SRGM scores in the TCGA-OV (n=306, log-rank test: P < 0.0001) **(D)**, GSE13876 (n=415, log-rank test: P = 0.0040) **(E)**, GSE17260 (n=110, log-rank test: P = 0.0098) **(F)**, GSE19829 (n=29, log-rank test: P = 0.071) **(G)**, and GSE26712 (n=195, log-rank test: P = 0.0058) **(H)** cohorts. **(I)** Univariate and multivariate Cox regression analyses were performed to explore the prognostic value of the SRGs and clinicopathological features.

We employed 80 combinations of ten machine learning algorithms (RSF, Enet, Lasso, Ridge, stepwise Cox, CoxBoost, plsRcox, SuperPC, GBMand survival-SVM) to assess the TCGA-OV cohort as the training set. Validation was conducted on the independent datasets GSE13876, GSE17260, GSE19829 and GSE26712 (**Figure 2A**, **Supplementary Table S5**). On the basis of the average C-index ranking, we identified the algorithmic pattern (Enet [α=0.2] and StepCOX [both]) with the highest average C-index (0.654). For Enet [α=0.2], the optimal λ was determined via the leave-one-out cross-validation (LOOCV) framework when the partial likelihood deviance reached its minimum. Thirty-eight genes with coefficients greater than 0.01 were subjected to stepwise Cox proportional hazards regression, which identified a final set of 22 SRGs. This model, the SUMOylation-related gene model (SRGM), was used to compute SRGM scores for each patient according to the expression of these 22 SRGs (ten risk genes: *PI3*, *SOGA1*, *AUP1*, *CCDC80*, *FBLN1*, *TSC22D1*, *RPS12*, *LAMC1*, *RAB34 and MRPS12*; 12 protective genes: *HSP90AB1*, *LUC7L2*, *KLF5*, *CD44*, *SPON1*, *DAP*, *COA4*, *LAMC2*, *GNAS*, *MAGED2*, *MMP9* and *CD200*) and their weighted Cox model coefficients (**Figure 2B**, **Supplementary Table S6**). Risk factor association plots revealed that the high-SRGM score group had a shorter survival time and a higher mortality rate (**Figure 2C**).

Additionally, Kaplan-Meier (KM) curves across the training and validation sets consistently demonstrated that patients with high SRGM score exhibited poorer survival, indicating the strong prognostic value and broad applicability of these 22 SRGs (**Figures 2D-H**). We stratified patients from the TCGA-OV cohort into various subgroups on the basis of their clinical information. We found that patients with high SRGM scores presented poorer prognoses across patients grouped by age, postoperative residual tumor status, tumor division, tumor stage and tumor grade (**Supplementary Figure S3A**). Univariate and multivariate Cox regression analyses were conducted to assess the predictive value of the SRGM score and clinical factors (**Figure 2I**). SRGM scores emerged as the sole significant predictor of ovarian cancer outcomes in both Cox regression models.

With the rapid advancement of big data technologies, including high-throughput sequencing and machine learning, an increasing number of prognostic features have been identified to facilitate precise medical management of cancer patients (25). To comprehensively compare the performance of the SRGM with other models to predict survival among ovarian cancer patients, we compiled information on 50 published prognostic gene signatures and computed their respective C-index values (**Supplementary Figure S3B**, **Supplementary Table S7**). The SRGM consistently exhibited superior performance across all five independent datasets, underscoring its superior predictive accuracy and broad applicability (**Supplementary Table S8**). These findings also suggest that among all the SRGs, 22 SRGs comprising the SRGM have the most significant impact on ovarian cancer prognosis.

### 3.3 SUMOylation inhibition regulates the expression of key SRGs

**Figure 3:**
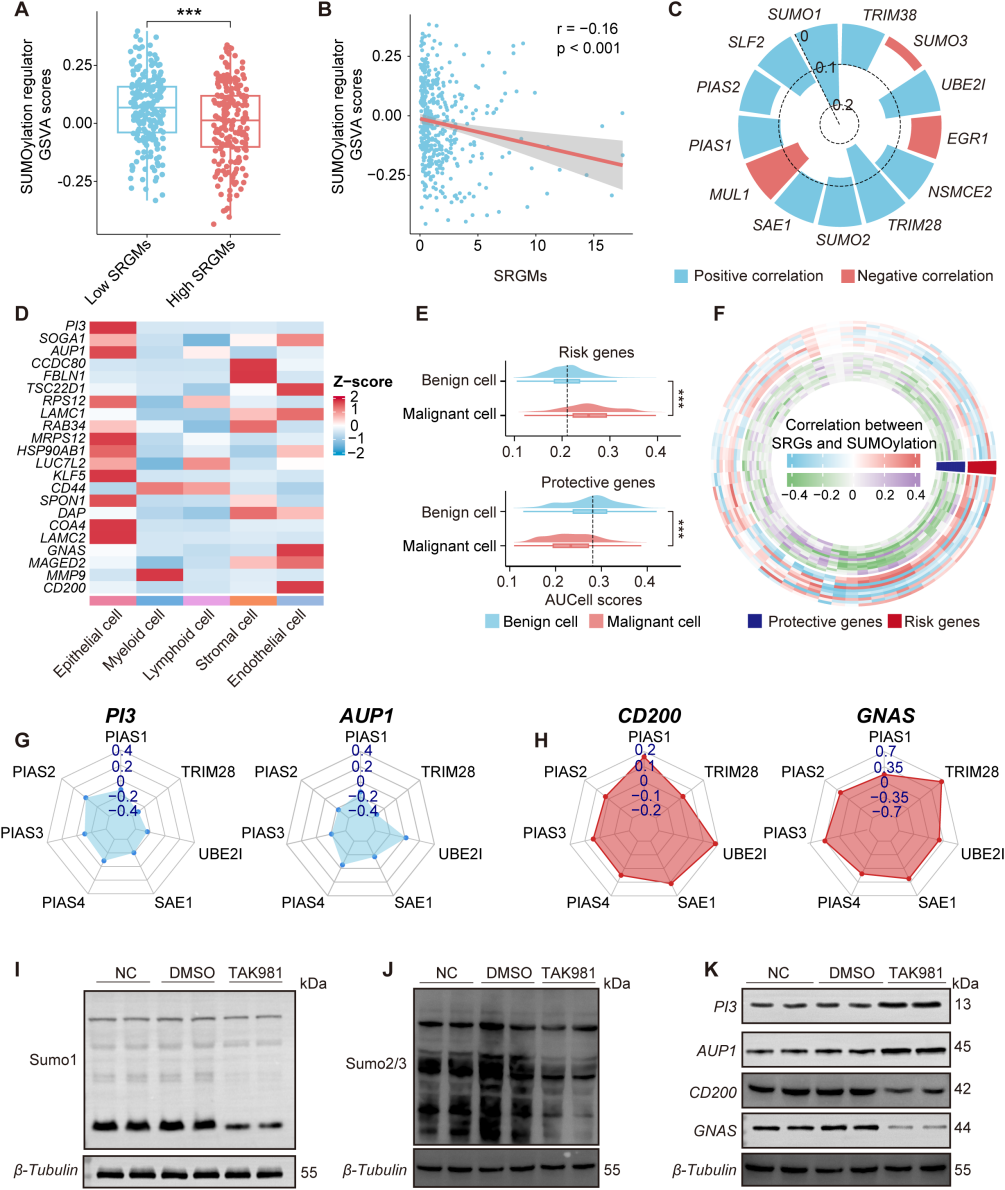
SUMOylation inhibition regulates the expression of key SRGs. **(A)** Boxplot comparing the GSVA scores of SUMOylation regulators between patients with high and low SRGM scores. **(B)** Scatterplots illustrating the correlation between GSVA scores of SUMOylation regulators and SRGM scores. **(C)** Circular bar plot showing Spearman correlation coefficients between the SRGM score and SUMOylation regulators expression, highlighting positive (red) and negative (blue) correlations. **(D)** Heatmap showing the distribution of the expression of 22 SRGs across five cell clusters. **(E)** Violin plots representing the differences in the expression of risk genes and protective genes between malignant and benign epithelial cells. **(F)** Ring heatmap showing Spearman correlations between SUMOylation regulators and key genes. Red indicates risk genes, and blue indicates protective genes. **(G, H)** Radar plot depicting the Spearman’s rank correlation coefficient between seven SUMOylation regulators and two risk genes (*PI3* and *AUP1*) **(G)** and two protective genes (*CD200* and *GNAS*) **(H)**. **(I-K)** Western blot showing the changes in SUMO1 **(I)**, SUMO2/3 **(J)**, *PI3*, *AUP1*, *CD200*, and *GNAS* **(K)** expression in primary ovarian cancer cells after treatment with DMSO or TAK981. Each experiment was independently repeated three times. ***, P < 0.001.

We first performed a differential analysis of the GSVA scores of SUMOylation regulators between patients with high and low SRGM scores in the TCGA-OV cohort (**Figure 3A**). The results indicated that patients with high SRGM scores exhibited significantly lower GSVA scores of SUMOylation regulators and SRGM scores were negatively correlated with these GSVA scores (**Figure 3B**). Additionally, SRGM scores showed a significant negative correlation with the expression of various key SUMOylation regulators (**Figure 3C**).

To further explore the potential link between the expression of the 22 SRGs comprising the SRGM and SUMOylation levels at the single-cell level, we conducted expression profiling across five distinct cell clusters and normalized the data based on Z-scores (**Figure 3D**). Our analysis revealed that epithelial cells presented relatively higher expression levels of these 22 SRGs than other cell clusters did. Next, we employed AUCell to assess the expression of risk genes and protective genes in both benign and malignant epithelial cells (**Figure 3E**). Compared to benign cells, malignant cells presented higher risk gene scores and lower protective gene scores, which is consistent with the notion that risk genes promote cancer progression, whereas protective genes inhibit it.

SUMOylation can impact downstream gene expression through various mechanisms, such as altering the activity and localization of transcription factors, influencing chromatin structure and regulating signaling pathways. Therefore, we sought to further investigate whether SUMOylation inhibition in ovarian cancer could significantly influence, or more specifically regulate the expression levels of certain key SRGs. Correlation analysis further revealed potential associations between 68 SUMOylation regulators and 22 SRGs (**Figure 3F**). We found that four key SRGs—two risk genes *PI3* and *AUP1* and two protective genes *CD200* and *GNAS*—are most significantly correlated with SUMOylation regulators. The expression of risk genes *PI3* and *AUP1* was negatively correlated with the expression of key SUMOylation regulators, particularly the genes encoding critical enzymes involved in the SUMOylation process such as *SAE1* and *UBE2I*, whereas the expression of protective genes *CD200* and *GNAS* was positively correlated with these regulators (**Figures 3G, H**, **Supplementary Figure S4A**). To validate our findings, *in situ* tumor samples were collected during surgery from ovarian cancer patients who had not undergone chemotherapy and primary cells were promptly extracted. We then inhibited overall SUMOylation in these primary cells via TAK-981 (5 µM/ml, 12h). The WB results demonstrated that the addition of TAK-981 led to decreased levels of SUMO1 and SUMO2/3 in primary ovarian cancer cells, increased the protein levels of *PI3* and *AUP1* and decreased the protein expression of *CD200* and *GNAS*. We also performed qPCR analysis using RNA extracted from frozen tumor tissues of mice treated with TAK-981 or vehicle control. The results showed that the mRNA levels of *PI3* and *AUP1* were significantly upregulated in the TAK-981 group, while the expression levels of *CD200* and *GNAS* were notably reduced, consistent with the results of the correlation analysis (**Figures 3I-K**, **Supplementary Figure S4B**).

These findings suggest that the expression levels of key SRGs in ovarian cancer, including *PI3*, *AUP1*, *CD200* and *GNAS*, can be regulated by global SUMOylation levels.

### 3.4 Association between key SRGs and epithelial cell functional pathways and chemoresistance

**Figure 4:**
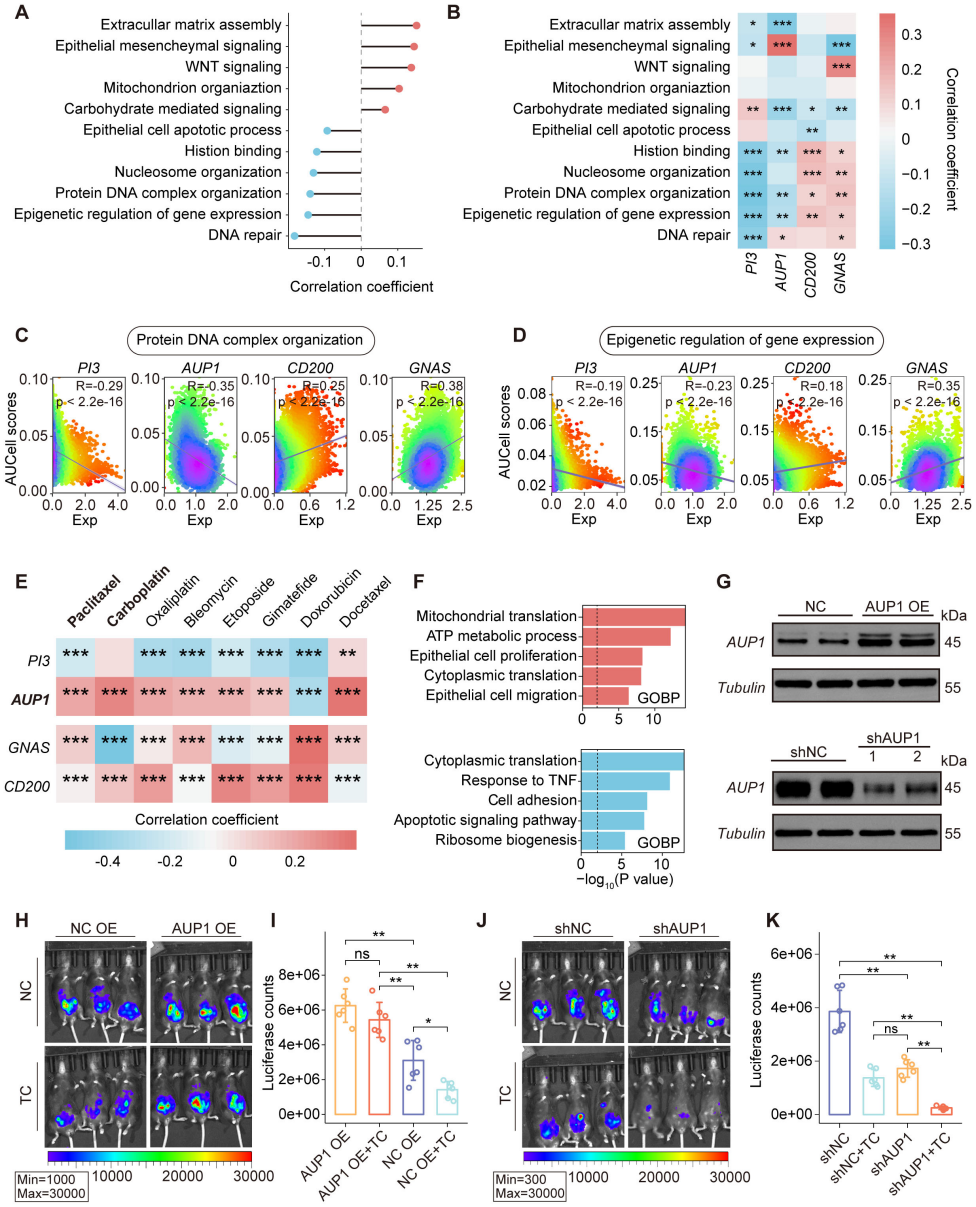
Association between the expression of key SRGs and epithelial cell functional pathways. **(A)** Correlations between SRGMs and GSVA scores of functional pathways in the TCGA-OV cohort. **(B)** Heatmap revealing the correlation between GSVA scores of functional pathways and the expression of *PI3*, *AUP1*, *CD200* and *GNAS*. **(C, D)** Scatterplot showing the expression of *PI3*, *AUP1*, *CD200* and *GNAS* in relation to the AUCell scores of the protein DNA complex organization pathway **(C)** and the epigenetic regulation of gene expression pathway **(D)** in epithelial cells. **(E)** Heatmap revealing the correlation between drug resistance scores and the expression of *PI3*, *AUP1*, *CD200* and *GNAS* in epithelial cell. **(F)** Horizontal bar graphs representing the most differential pathways. Functional enrichment of genes with higher (up, red) and lower (down, blue) expression in high-*AUP1* expression epithelial cells than in low-*AUP1* expression epithelial cells. **(G)** *AUP1* overexpression (top) and *AUP1* knockdown (bottom) was confirmed by Western blotting. **(H)** Bioluminescence images showing the tumor burden of C57BL/6 mice following inoculation with *AUP1* overexpression luciferase-tagged ID8 cells and subsequent treatment with TP. **(I)** The histogram showing the differences in tumor burden between the *AUP1* OE group and the negative control NC group following TP treatment. **(J)** Bioluminescence images showing the tumor burden of C57BL/6 mice following inoculation with sh*AUP1* luciferase-tagged ID8 cells and subsequent treatment with TP. **(K)** The histogram showing the differences in tumor burden between the sh*AUP1* group and the negative control NC group following TP treatment. ns., not significant; *, P < 0.05; **, P < 0.01; ***, P < 0.001.

To investigate the role of key SRGs in ovarian cancer, we performed a correlation analysis between the SRGM scores of each patient and the GSVA scores of tumor-associated functional pathways in TCGA-OV cohort (**Figure 4A**). The results revealed that SRGM scores were positively correlated with the activity of pathways such as epithelial-mesenchymal signaling and extracellular matrix assembly, while negatively correlated with the GSVA scores of various epigenetic processes, including histone binding and protein-DNA complex organization. Correlation analysis between the four key SRGs and these functional pathways revealed that the expression of risk genes *PI3* and *AUP1* was negatively correlated with the activity of these epigenetic processes, consistent with the findings based on SRGM scores. In contrast, the expression of protective genes *CD200* and *GNAS* showed an opposite trend (**Figure 4B**). These findings were further validated in single-cell sequencing data (**Figures 4C, D**).

Epigenetic dysregulation is closely associated with the proliferation, invasion and chemoresistance of ovarian epithelial cells. Chemoresistance is one of the major factors contributing to poor prognosis in ovarian cancer patients. Using Beyondcell, a computational tool for predicting drug sensitivity in single-cell RNA-seq data, we assessed the resistance scores of each epithelial cell to commonly used chemotherapeutic agents in ovarian cancer and analyzed their correlation with gene expression (**Figure 4E**). We found that *AUP1* expression was significantly positively correlated with resistance scores of paclitaxel and carboplatin. Further, based on *AUP1* expression, we classified epithelial cells into two groups and performed differential enrichment analysis (**Figure 4F**). The results showed that epithelial cells with high *AUP1* expression exhibited enhanced activities in epithelial cell proliferation, while exhibiting reduced activities in response to TNF.

Given the close association between *AUP1* and epithelial cell proliferation and chemoresistance, we established the peritoneal ovarian cancer mouse model and conducted two groups of experiments. ID8 cells transfected with negative control (NC, overexpression or knockdown), *AUP1* overexpression (*AUP1* OE), or *AUP1* knockdown (sh*AUP1*) plasmids were intraperitoneally injected into mice (**Figure 4G**, **Supplementary Figure S4C**). The mice were treated with either the carboplatin-paclitaxel (TC) regimen or saline. We observed that mice injected with *AUP1* OE ID8 cells exhibited a significantly higher tumor burden than the NC OE group. Although the NC OE group showed a marked reduction in tumor burden following TC treatment, the same regimen did not significantly reduce the tumor burden in *AUP1* OE mice (**Figures 4H, I**). These findings suggest that *AUP1* overexpression not only accelerates tumor progression but also promotes chemotherapy resistance. Conversely, mice injected with sh*AUP1* ID8 cells exhibited a significantly lower tumor burden compared to the shNC group, with further reduction after TC treatment (**Figures 4J, K**).

Overall, these findings suggest that the expression of key SRGs, including *PI3*, *AUP1*, *CD200* and *GNAS*, is closely associated with the epigenetic regulation of epithelial cells. Among them, AUP1 overexpression may serve as a risk factor for chemoresistance and targeting AUP1 could potentially act as a therapeutic strategy to improve chemotherapy outcomes.

### 3.5 CD8^+^ cytotoxic T cells with high *CCDC80* expression exhibits impaired anti-tumor function

The tumor immune microenvironment (TIME) refers to a complex network of tumor cells, immune cells, stromal components and secreted factors that plays a pivotal role in tumorigenesis, progression, immune evasion and therapeutic response (26). Interactions between immune cells and epithelial cells are bidirectional, mediated through mechanisms such as signal secretion and receptor activation, cell-cell contact and metabolic crosstalk (27).

**Figure 5:**
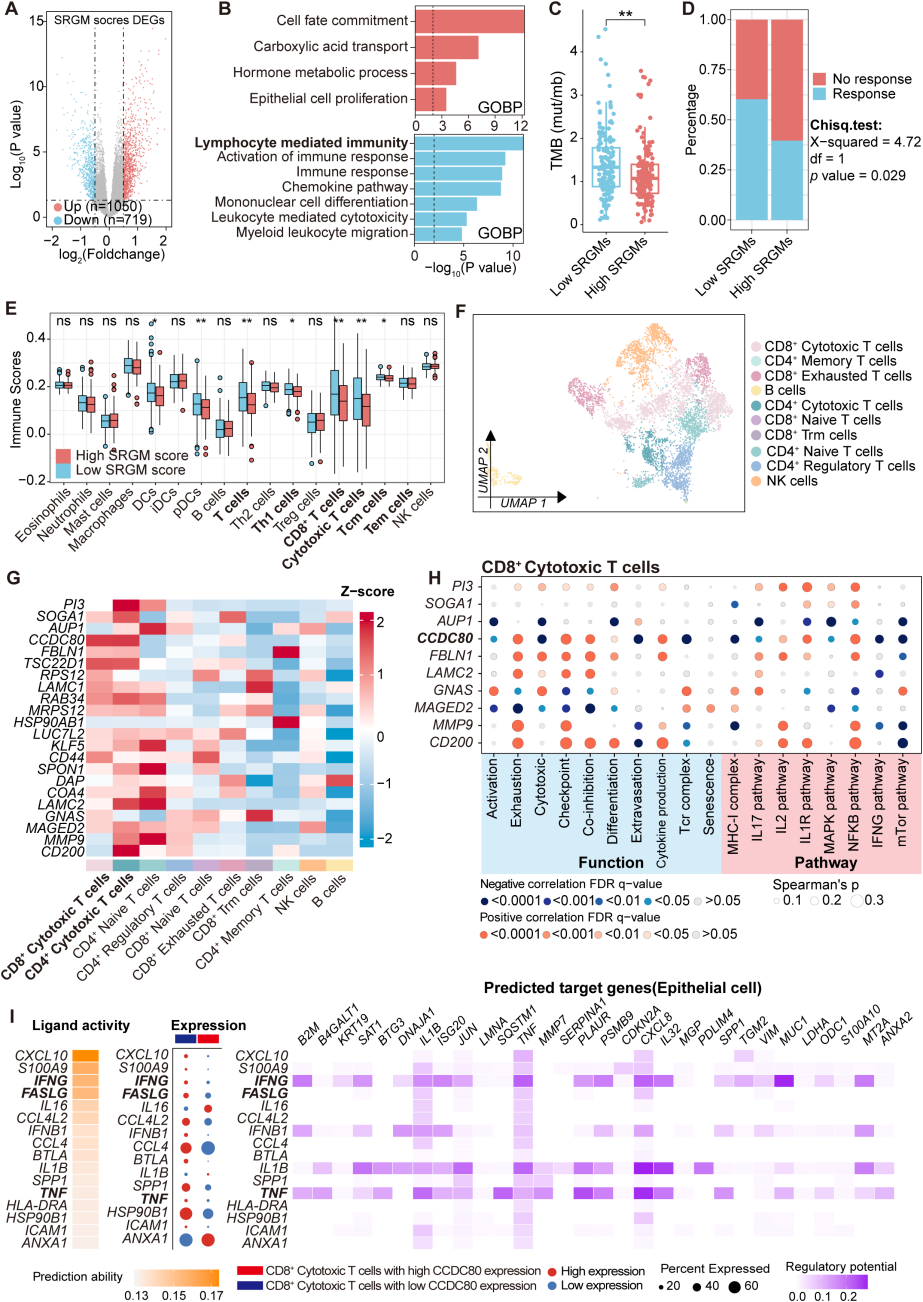
CD8^+^ cytotoxic T cells with high *CCDC80* expression exhibited reduced anti-tumor function activities. **(A)** Volcano plot comparing differentially expressed genes between patients with high and low SRGM scores in the TCGA-OV cohort. **(B)** Horizontal bar graphs representing the most differential pathways. Functional enrichment of genes with higher (up, red) and lower (down, blue) expression in high-SRGM score patients than in low-SRGM score patients. **(C)** Comparison of TMB in the high- and low-SRGM score groups (n=306). **(D)** Predicted differences in the immunotherapy response between the high- and low-SRGM score groups according to the TIDE algorithm. **(E)** Comparisons of the abundances of 17 immune cells in the high- and low-SRGM score groups are shown in boxplots. **(F)** Lymphoid cells are reclustered and presented in UMAP plots. **(G)** Heatmap showing the distribution of the expression of 22 SRGs across the clusters. **(H)** Heatmap illustrating the Spearman correlation between the expression of SRGs (top five risk genes and top five protective genes) and the AUCell scores of cell functions, pathways, and metabolism across CD8^+^ cytotoxic T-cell clusters. **(I)** NicheNet was employed to analyze ligand and receptor expression, with the aim of elucidating how changes in *CCDC80* expression in CD8^+^ cytotoxic T cells regulate intercellular communication patterns between epithelial cells and CD8^+^ cytotoxic T cells. ns., not significant; *, P < 0.05; **, P < 0.01.

To delve deeper into the association between key SRGs and TIME in ovarian cancer, we stratified patients from the TCGA-OV cohort on the basis of the SRGM score and conducted differential analysis, revealing 1,769 differentially expressed genes (**Figure 5A**). GO enrichment analysis of these genes revealed that immune-related pathways, such as lymphocyte-mediated immunity and leukocyte-mediated cytotoxicity, were significantly enriched among the downregulated genes (**Figure 5B**). Moreover, patients with high SRGM scores exhibited reduced tumor mutation burden (TMB) (**Figure 5C**). These findings suggest that patients with higher SRGM scores may experience suppressed antitumor immune function. Using the TIDE tool, we found that the high-SRGM score group presented a greater proportion of patients in this group demonstrated resistance to immunotherapy (**Figure 5D**).

We then performed single-sample gene set enrichment analysis (ssGSEA) to calculate immune scores for 17 common immune cell markers in each patient, which represented the abundance of these immune cells (**Figure 5E**, **Supplementary Table S9**). We found that lymphocyte populations, including T cells, CD8^+^ T cells and CD8^+^ cytotoxic T cells, were significantly lower in patients with high SRGM scores.

Single-cell data from ovarian cancer patients was utilized to perform a detailed analysis of the relationship between key genes and lymphocyte function. Lymphoid cells were reclustered into four main clusters: CD8^+^ T cells, CD4^+^ T cells, NK cells and B cells (**Supplementary Table S10**). Additionally, CD4^+^ T cells were subdivided into four clusters: CD4^+^ cytotoxic T cells, CD4^+^ naive T cells, CD4^+^ regulatory T cells and CD4^+^ memory T cells (**Supplementary Table S11**). Similarly, CD8^+^ T cells were divided into four clusters: CD8^+^ cytotoxic T cells, CD8^+^ naive T cells, CD8^+^ exhausted T cells and CD8^+^ Trm cells (**Figure 5F**, **Supplementary Figure S5A**, **Supplementary Table S12**) (17). We conducted expression profiling across ten distinct cell clusters. We observed that, compared with other subclusters of T cells, CD8^+^ cytotoxic T cells and CD4^+^ cytotoxic T cells presented the highest relative expression levels of 22 SRGs (**Figure 5G**). Additionally, CD8+ cytotoxic T cells constituted the largest proportion of all lymphocytes (**Supplementary Figure S5B**).

Previous studies have demonstrated that cytotoxic CD8^+^ T cells form the backbone of cancer immunotherapy and that their dysfunction can lead to poor responses to immunotherapy in solid tumors (28, 29). Therefore, we then focused on whether SRGs could influence the function of cytotoxic CD8^+^ T cells. The top five risk genes and five protective genes were selected and correlation analysis was performed between their expression and key functional and metabolic pathway AUCell scores in CD8^+^ cytotoxic T cells (**Figure 5H**, **Supplementary Table S13**). Importantly, the expression of *CCDC80* was negatively correlated with activation and cytotoxicity but positively correlated with exhaustion and coinhibition. Subsequently, using Nichenet, we found that elevated expression of *CCDC80* was found to reduce the activity of three critical signaling pathways involved in CD8^+^ T cell cytotoxic function: the FASLG, TNF and IFN-γ ligand–receptor pathways, leading to alterations in the expression of *CXCL8* and *MUC1* in epithelial cells (**Figure 5I**).

Overall, these results suggest that CD8^+^ cytotoxic T cells with high *CCDC80* expression exhibit inhibited cytotoxicity activities against tumor cells, which may contribute to immune evasion and promote an immunosuppressive TIME.

### 3.6 Therapeutic potential and prognostic value of key SRGs in ovarian cancer patients

**Figure 6:**
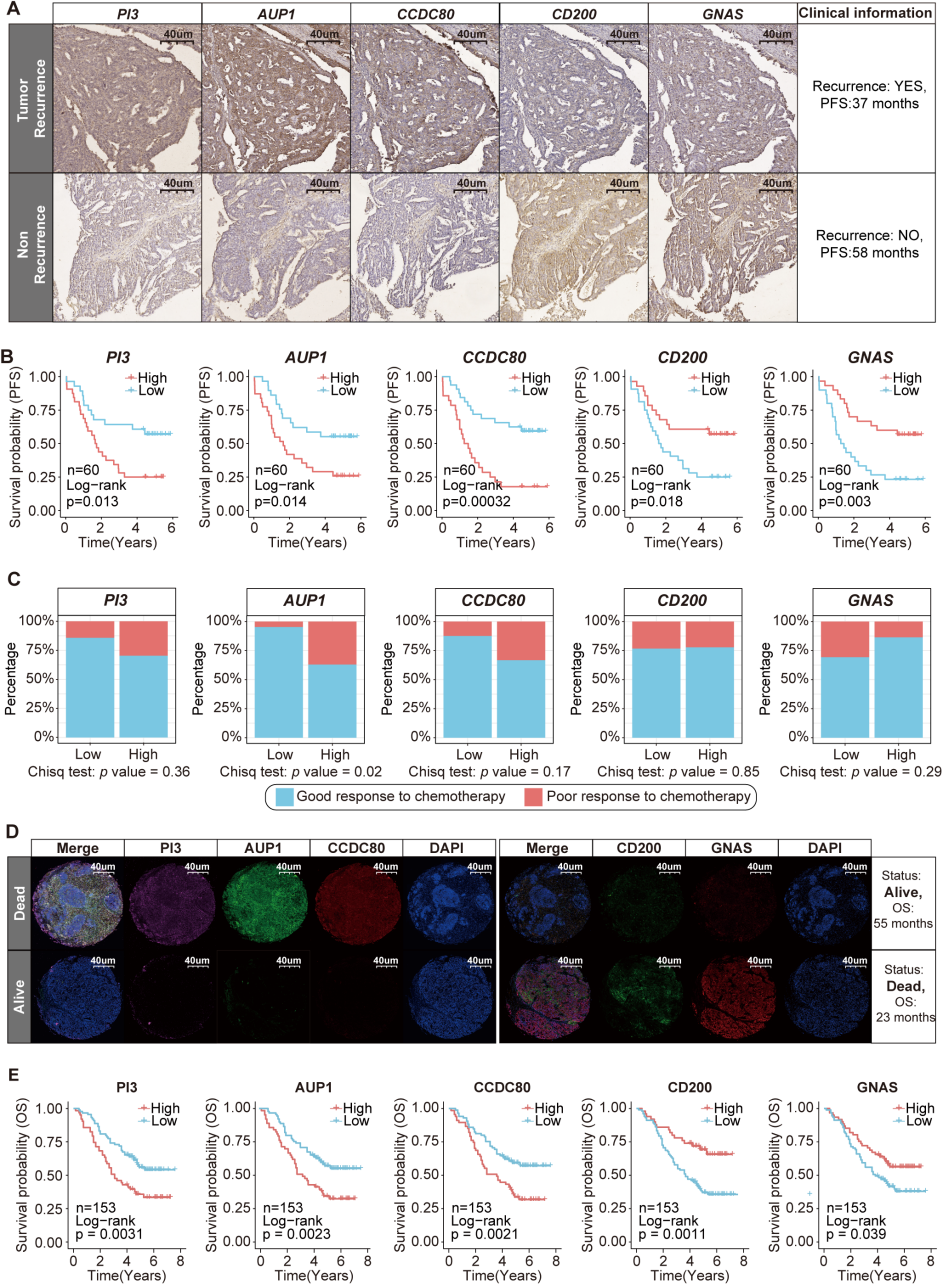
Clinical cohort validation of the prognostic value and therapeutic potential of five key SRGs. **(A)** Immunohistochemical staining of *PI3*, *AUP1*, *CCDC80*, *CD200* and *GNAS* in patients with and without recurrence. **(B)** Kaplan–Meier curves of PFS according to the expression levels of *PI3*, *AUP1*, *CCDC80*, *CD200* and *GNAS* (n=60). **(C)** The stacked bar chart illustrates the distribution differences in the chemotherapy response between the high- and low-expression groups of five key SRGs (n=60). **(D)** Immunofluorescence staining revealed differences in the expression of *PI3*, *AUP1*, *CCDC80*, *CD200* and *GNAS* between deceased and surviving patients. **(E)** Kaplan–Meier curves of OS according to the expression levels of *PI3*, *AUP1*, *CCDC80*, *CD200* and *GNAS* (n=153).

After comprehensively analyzing the effects of key SRGs on ovarian epithelial cells and the TIME, we aimed to validate the prognostic effect of these five candidate SRGs *AUP1*, *PI3*, *CD200*, *GNAS* and *CCDC80*. We collected FFPE samples from 60 patients who underwent surgical treatment at West China Second University Hospital between December 2017 and June 2019, tracking their recurrence events over a five-year follow-up period (**Supplementary Figure S6A**, **Supplementary Table S14**). We performed IHC staining for these five SRGs (**Supplementary Figure S6A**). The results revealed that the expression levels of *PI3*, *AUP1* and *CCDC80* were markedly increased in patients with recurrence than in those without recurrence, whereas the expression levels of *CD200* and *GNAS* were lower in patients with recurrence (**Figure 6A**, **Supplementary Figure S8A**). Additionally, the Kaplan–Meier analysis indicated that patients with high expression levels of risk genes *PI3*, *AUP1* and *CCDC80* presented poorer progression-free survival (PFS) within five years, whereas those with high expression of protective genes *CD200* and *GNAS* presented better PFS outcomes (**Figure 6B**).

The established standard chemotherapy regimen currently consists of a combination of carboplatin and paclitaxel. Among the 60 patients included in the study, 50 received this carboplatin–paclitaxel regimen. Among these 50 patients, three experienced disease progression during chemotherapy, while eight patients relapsed within six months after completing treatment. Both scenarios are classified as indicative of a poor response to chemotherapy (2). We found that patients with high *AUP1* expression were more likely to experience a poor response to chemotherapy (**Figure 6C**).

Subsequently, we aimed to explore the association between the five key SRGs and overall survival (OS) in another clinical cohort. This cohort consists of 153 ovarian cancer patients in total who underwent surgical treatment without neoadjuvant therapy at West China Second University Hospital between January 2014 and January 2017, with a maximum follow-up period of 91 months (**Supplementary Figure S6B**, **Supplementary Table S15**). FFPE samples from these patients were used to construct tissue microarrays and subjected to immunofluorescence staining (**Supplementary Figures S6B, C**). The results showed that compared to surviving patients, deceased patients presented higher immunofluorescence staining intensity for risk genes *PI3*, *AUP1* and *CCDC80* and lower staining intensity for protective genes *CD200* and *GNAS*, with statistically significant differences (**Figure 6D**, **Supplementary Figure S8B**). Furthermore, patients with high *AUP1* expression were older and presented with more advanced disease stages, highlighting the close association between *AUP1* and clinical risk factors in ovarian cancer patients (**Supplementary Figure S8C**). Most importantly, we found that patients with high expression of the risk genes *PI3*, *AUP1* and *CCDC80* had shorter survival times, whereas those with elevated expression of the protective genes *CD200* and *GNAS* demonstrated better clinical prognosis (**Figure 6E**).

On the whole, our study identified five key SRGs, including the risk genes *AUP1*, *PI3* and *CCDC80*, as well as the protective genes *CD200* and *GNAS* and elucidated their prognostic potential in ovarian cancer.

## 4 Discussion

Ovarian cancer is the third most common gynecologic malignancy worldwide but has the highest mortality rate among these cancers (30). Treatment primarily relies on debulking surgery to achieve no residual disease and platinum-based chemotherapy. While these treatments have led to a reduction in OC-related mortality to some extent, patient outcomes remained unfavorable, highlighting the urgent need to develop new develop new prognostic signatures and molecular biomarkers (7).

SUMOylation, a posttranslational modification, has been implicated in the initiation and progression of various cancers. SUMOylation modulates gene expression through multiple mechanisms, including the alteration of the activity, localization and stability of transcription factors, coactivators and corepressors (12, 31). In this study, we observed the unique characteristic of global SUMOylation in ovarian cancer. Although several studies have demonstrated the therapeutic potential of SUMOylation-targeting drugs, such as TAK981 (20), ginkgolic acid (32) and kerriamycin B (33), in cancers such as pancreatic and liver cancer, our findings suggest that the therapeutic efficacy of SUMOylation inhibition in ovarian cancer is suboptimal. Previous research has demonstrated that SUMOylation is essential for maintaining adult ovarian homeostasis and preserving normal function in conditional knockout mouse models (34). In contrast, PIAS1 inhibition leads to reduced SUMOylation, which, through activation of the TGFβ pathway, promotes EMT, increasing tumor cell survival and metastasis (11). Additionally, low levels of SUMOylation have been shown to increase HIF1-dependent VEGF expression, thereby promoting tumor angiogenesis (35). Tumor angiogenesis facilitates cancer metastasis by supplying oxygen, nutrients and routes for dissemination (36). Stergios J. et al. reported that distant metastatic lesions exhibit lower levels of SUMOylation, whereas primary tumors tend to have higher expression (37). Therefore, considering the unique characteristics of SUMOylation in various cancers, intervening in SUMOylation levels in ovarian cancer patients may not only disrupt the homeostasis of normal cells but also drive cancer cells to adopt a more aggressive and metastatic phenotype. Thus, targeting SUMOylation-regulated downstream targets, SRGs, may represent a more effective strategy.

This study is unique in that it involves 80 combinations of ten distinct algorithms to construct a prognostic model SRGM and identify 22 SRGs closely associated with ovarian cancer survival and regulated by SUMOylation. The SRGM model, composed of these 22 SRGs, demonstrated superior performance across all five independent datasets compared to 50 published prognostic models, further confirming the close association between these 22 SRGs and prognosis. Through single-cell analysis, we further identified five potential prognostic biomarkers associated with the ovarian cancer cell functional pathway and TIME: three risk genes *PI3*, *AUP1* and *CCDC80* and two protective genes *CD200* and *GNAS*.

In epithelial cells, low SUMOylation levels drive the upregulation of risk genes *PI3* and *AUP1* while downregulating protective genes *CD200* and *GNAS*. PI3, also known as Elafin, is a member of the whey acidic protein four-disulfide core (WFDC) family (38). This protein plays a crucial role in modulating inflammatory responses, protecting against tissue damage, and inhibiting elastase-mediated proteolysis. It is significantly involved in both the physiology and pathology of the reproductive system. Emerging evidence suggests that PI3 concentration and subcellular localization may serve as potential biomarkers for assessing cervical cancer progression (39). Notably, in ovarian cancer, Lu et al. demonstrated that elevated PI3 expression correlates with poorer overall survival, which is consistent with our findings (40). CD200, also known as OX-2, is a single-pass type I membrane glycoprotein belonging to the immunoglobulin superfamily (IgSF) (41). Although numerous studies have demonstrated that CD200 is upregulated in various types of cancer and is associated with immunosuppression, there is also evidence indicating that CD200 may exert anti-tumor effects (42, 43). These effects are thought to arise from its ability to suppress tumor-promoting inflammation, inhibit angiogenesis, and limit the expansion of tumor-associated myeloid cells (TAMCs) (44, 45). In a B16 melanoma model, inoculation with CD200-positive B16 melanoma cells inhibited tumor formation and growth in C57BL/6 mice and significantly reduced the formation of metastatic foci in the lungs (46). GNAS (guanine nucleotide-binding protein, alpha-stimulating) is an imprinted gene located on the q arm of chromosome 20 with a complex genomic locus (47). Its major product, Gsα, consists of a Ras-like domain with GTPase activity and an α-helical domain. Gsα plays a vital role in transducing signals from G protein-coupled receptors (GPCRs), thereby activating downstream effectors involved in diverse cellular processes (48). Proper functioning of Gsα is essential for maintaining normal cellular responses to external stimuli, and dysregulation of Gsα signaling has been implicated in various diseases, including endocrine disorders and cancers (49, 50). Notably, loss or reduced expression of *GNAS* has been associated with increased tumor aggressiveness and poor survival in medulloblastoma (51). In the present study, both univariate Cox regression analysis and integrated machine learning approaches identified *CD200* and *GNAS* as protective biomarkers in ovarian cancer. However, the mechanisms through which *CD200* and *GNAS* influence ovarian cancer prognosis remain unclear and require further investigation through *in vitro* and *in vivo* experiments.

Correlation analysis shown that the expression level of *PI3*, *AUP1*, *CD200* and *GNAS* is closely associated with the activities of epigenetic regulation processes, such as histone binding and the organization of protein-DNA complexes. Epigenetic modifications in ovarian cancer led to chromatin structural changes that enhance the expression of genes involved in DNA repair, differentiation, angiogenesis and metastasis, thereby promoting tumorigenesis (52). Another study found that high levels of histone-modifying enzymes, such as the H3K9 methyltransferase G9a, are associated with advanced, high-grade, serous ovarian cancer and shorter survival in ovarian cancer patients (53). Shang et al. demonstrated that small molecule epigenetic inhibitors modulate key cellular signaling pathways, including NF-κB, TGFβ and WNT signaling, as well as major metabolic pathways, such as fatty acid metabolism and the TCA cycle, ultimately reducing platinum resistance in ovarian cancer cell lines (54). These findings suggest that reduced SUMOylation levels may regulate the expression of *PI3*, *AUP1*, *CD200* and *GNAS* in ovarian cancer cells, thereby influencing cancer cell survival, proliferation, metastasis and chemoresistance by modulating cell functions and metabolism.

Postoperative chemotherapy is crucial for improving the overall survival rate of ovarian cancer patients. The current consensus standard for chemotherapy involves a combination of carboplatin and paclitaxel. Our study revealed that overexpression of the SUMOylation-related gene *AUP1* is associated with the resistance of ovarian cancer cells to paclitaxel and carboplatin chemotherapy regimens. AUP1 (ancient ubiquitous protein 1) is an endoplasmic reticulum (ER)-associated protein (55) and more recently, it has been found to be abundantly expressed on the surface of lipid droplets (LDs) (56). Elevated levels of AUP1 enhance the ability of cells to efficiently degrade soluble terminally misfolded proteins, such as RI332-HA and NHK, leading to further accumulation of lipid droplets within the cell (57). Abnormal accumulation of lipid droplets (LDs) in tumor cells can promote the phosphorylation and degradation of E-cadherin or alter the palmitoylation levels of Wnt, thereby activating the Wnt/β-catenin signaling pathway to induce EMT in tumor cells (58). EMT can effectively induce ovarian cancer cell proliferation, invasion and drug resistance (59). Therefore, *AUP1* expression levels in cancer cells could serve as biomarkers for predicting ovarian cancer sensitivity to postoperative chemotherapy with paclitaxel and carboplatin. Targeting *AUP1* could potentially act as a novel therapeutic strategy improve the patient outcomes.

*CCDC80* (Coiled-Coil Domain Containing 80), also known as *DRO1* or *SSG1*, is located at 3q13.2 (60). Previous studies have demonstrated that elevated *CCDC80* expression is associated with reduced immune infiltration, poor response to immunotherapy, and worse prognosis in colorectal cancer and muscle-invasive bladder cancer, suggesting a strong association between aberrantly high *CCDC80* expression and the formation of an immunosuppressive tumor microenvironment (61, 62). To date, most research on *CCDC80* has focused on its expression and functional characteristics in tumor cells. For example, deletion of *CCDC80* impairs the growth-inhibitory effect mediated by LATS1/2 (Hippo pathway kinases) deficiency in MC38 cells, and silencing the immune-infiltration-associated gene *CCDC80* suppresses malignant progression and tumorigenicity in gastric cancer (63, 64). However, the expression pattern and functional role of *CCDC80* in immune cells remain largely unexplored. Our study revealed that the CD8^+^ cytotoxic T cells with high SRG *CCDC80* expression exhibit inhibited the antitumor immune functions. CD8^+^ cytotoxic T cells play crucial roles in the adaptive immune system, serve as the primary effectors in the antitumor immune response and form the foundation of cancer immunotherapy (29). Currently, progress in immunotherapy for ovarian cancer remains slow. Randomized phase III studies evaluating the addition of ICI monotherapy to standard cytotoxic chemotherapy in both first-line [e.g., JAVELIN 100 (65) and IMagyn050 studies (66)] and relapsed disease settings [e.g., JAVELIN 200 (67) and ATALANTE/ov29 trials (68)] have shown no benefit, failing to meet PFS and OS endpoints. The efficacy of ICI therapy can be improved by reinvigorating tumor-specific CD8^+^ T cells, as evidenced both *in vitro* and in murine models (69). Targeted inhibition (such as gene therapy) to specifically reduce *CCDC80* expression in CD8^+^ cytotoxic T cells, thereby reactivating their antitumor immune activity, has the potential to enhance the response of ovarian cancer patients to ICI treatment.

This study has several limitations. First, although we observed that inhibition of SUMOylation alters SRG expression at both the transcriptomic and proteomic levels, the direct mechanistic link between SUMOylation and these genes remains unclear. Second, although we identified SRGs involved in tumor progression and immune evasion, the underlying molecular mechanisms by which these genes modulate specific signaling pathways remain incompletely understood and warrant further mechanistic investigation. Additionally, the expression profiles and functional characteristics of these genes in epithelial and immune cells still need to be validated through further *in vivo* and *in vitro* experiments. Third, despite validation using an ID8 murine model and clinical samples from 213 ovarian cancer patients, the overall sample size may still be inadequate to fully capture the biological variability and rare genetic subtypes present in diverse patient populations. Finally, the therapeutic potential of AUP1 inhibition is promising, but further preclinical and clinical trials are necessary to evaluate its safety, efficacy, and potential off-target effects in humans. Addressing these limitations in future studies will be critical to enhancing the robustness and translational applicability of our findings.

Finally, we validated the aberrant expression of these five SRGs in 213 ovarian cancer patient samples from West China Second Hospital. Survival analysis further established their prognostic potential.

## 5 Conclusion

Through integrated machine learning and single-cell analysis, we identified five prognostic-associated SRGs (*PI3*, *AUP1*, *CD200*, *GNAS* and *CCDC80*) regulated by global SUMOylation levels in ovarian cancer. These SRGs are closely associated with ovarian cancer’s function, metabolism and TIME and may serve as potential biomarkers for predicting patient prognosis. Among them, we identified *AUP1*, whose overexpression may be a risk factor for postoperative chemoresistance. These findings have potential to improve patient outcomes and contribute to the ongoing advancement of precision medicine in oncology.

## Data Availability

The original contributions presented in the study are included in the article/**Supplementary Material**. Further inquiries can be directed to the corresponding author.
