# Supplementary material for: Integrated machine learning and single-cell analysis reveal the prognostic and therapeutic potential of SUMOylation-related genes in ovarian cancer
Source: Front Immunol. 2025 Jun 4;16:1577781. doi: 10.3389/fimmu.2025.1577781 (PMC12174102; doi:10.3389/fimmu.2025.1577781)
Supplement: Supplementary file 1 [file DataSheet1.pdf]

Supplementary Material

1 Supplementary Figures and Tables

1.1 Supplementary Figures

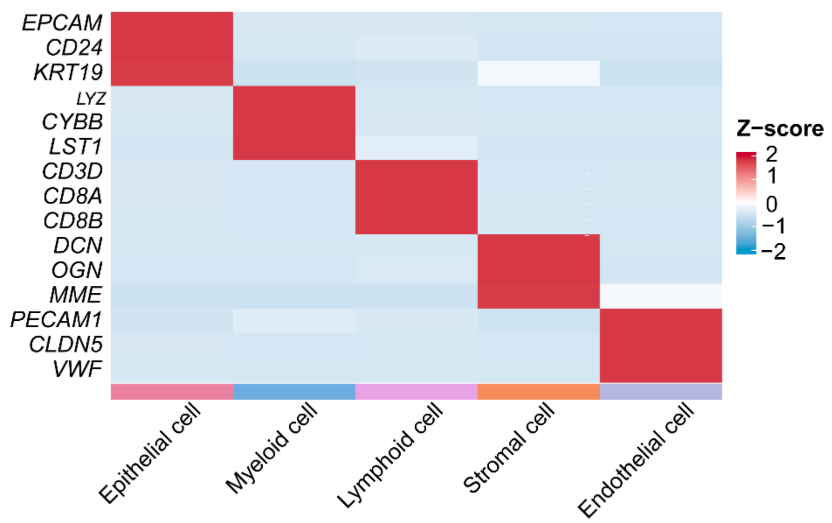

Supplementary Figure 1. Cell type-specific marker gene expression z score heatmap with cell types on the x-axis, and marker genes on the y-axis.

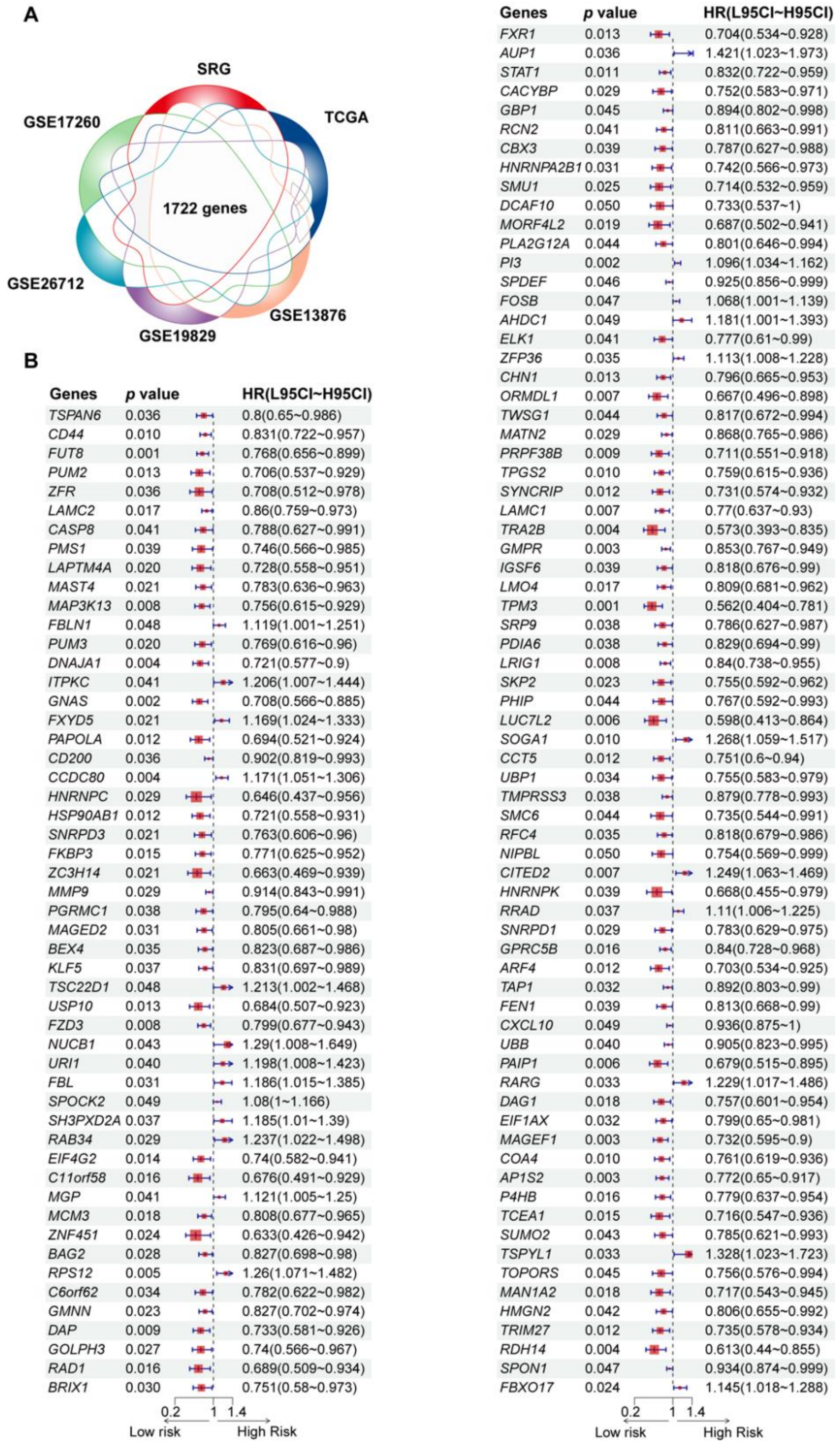

Supplementary Figure 2. SRGs were screened on the basis of TCGA and GEO data. A. Venn diagram showing the overlap between the SRGs among the five datasets. B. Forest plot displaying 124 SRGs with p values less than 0.05 from the univariate Cox analysis.

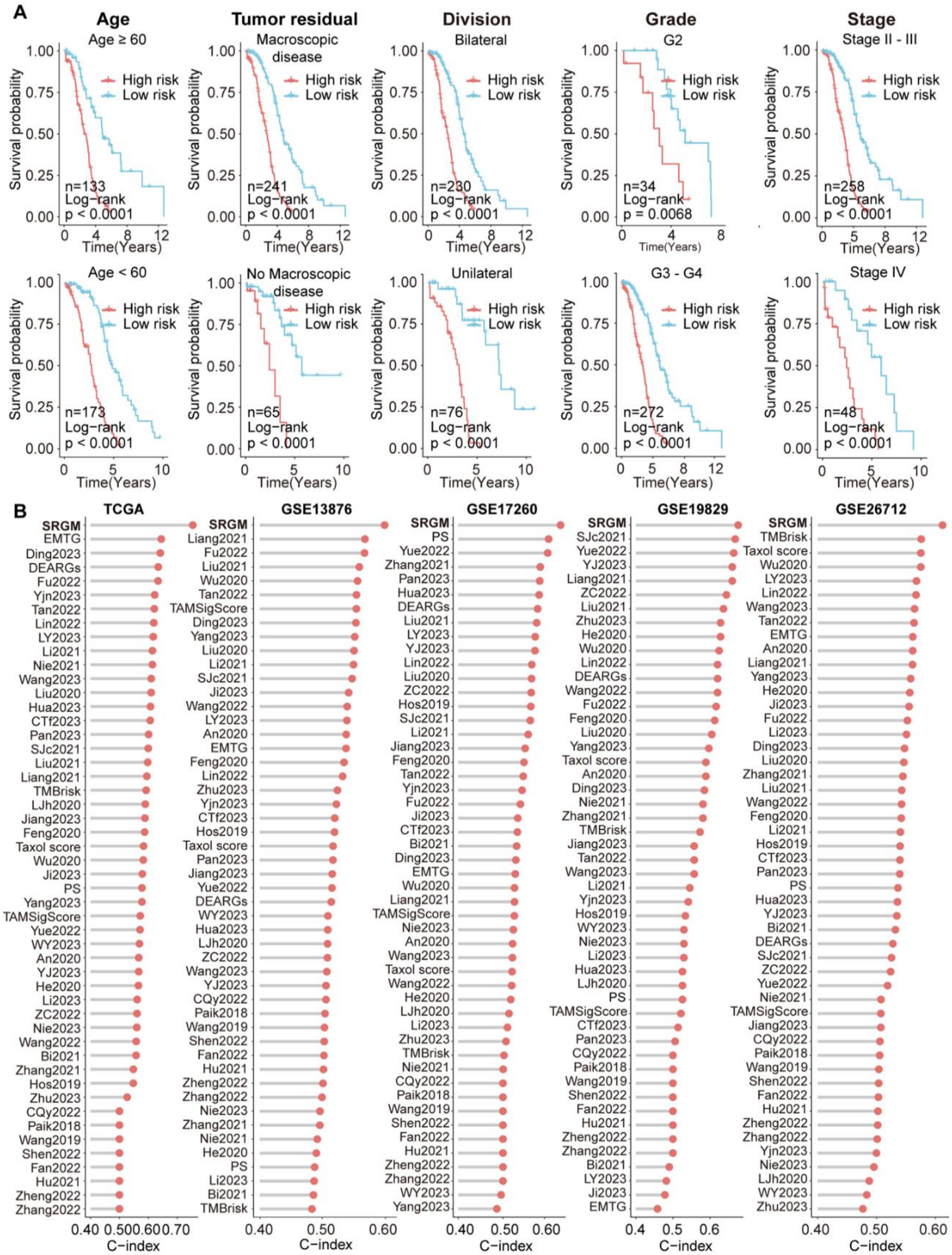

Supplementary Figure 3. Robust predictive ability of the SRGM score. A. Kaplan–Meier survival curves from subgroup analyses on the basis of various clinical variables, including age, residual tumor, tumor division, tumor grade, and tumor stage. B. C-index analysis of the SRGM signature and 50 published signatures in the TCGA-OV, GSE13876, GSE17260, GSE19829, and GSE26712 cohorts. P values were determined by log-rank test (A).

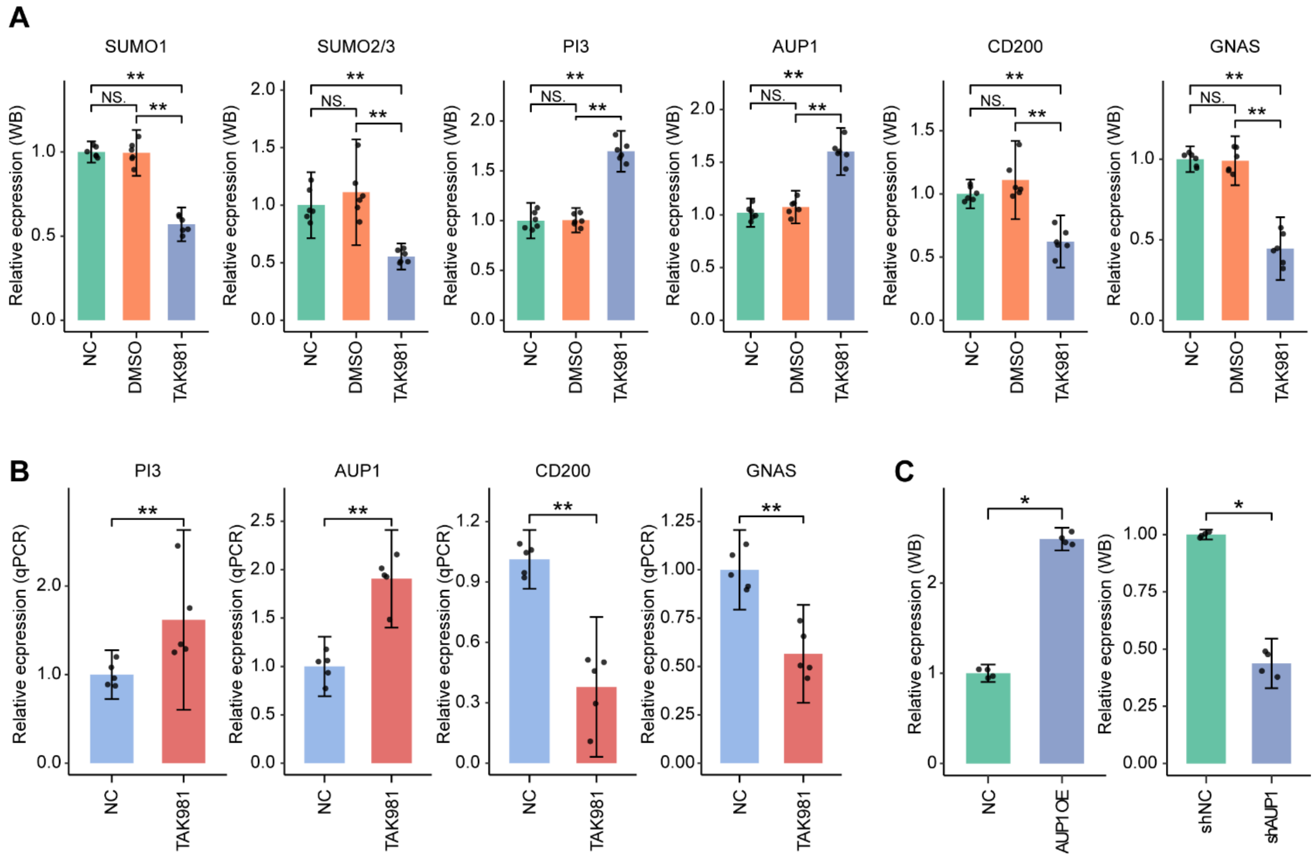

Supplementary Figure 4. SUMOylation inhibition regulate the expression of SRGs A. Western blot showing the changes in SUMO1, SUMO2/3, *PI3*, *AUP1*, *CD200*, and *GNAS* expression in primary ovarian cancer cells after treatment with DMSO or TAK981. Each experiment was independently repeated three times. **B.** The expression levels of *PI3* and *AUP1* were significantly upregulated, whereas those of *CD200* and *GNAS* were markedly downregulated in mouse tumor tissues following TAK981 treatment. **C.** *AUP1* overexpression (left) and *AUP1* knockdown (right) was confirmed by Western blotting.

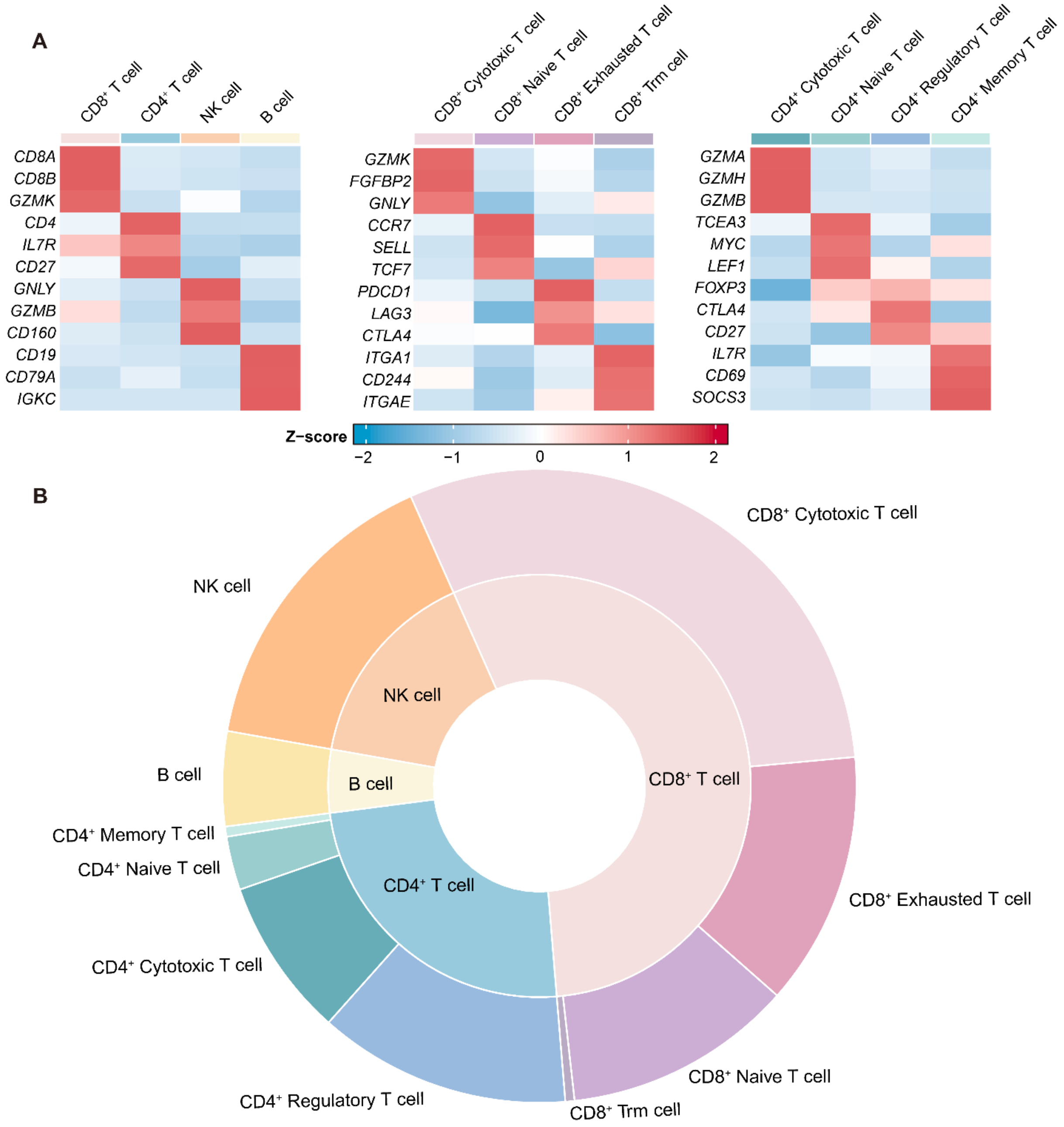

Supplementary Figure 5. Composition of lymphocytes in the immune microenvironment of ovarian cancer. A. Cell type-specific marker gene expression Z score heatmap with cell types on the x-axis and marker genes on the y-axis. The data were normalized and scaled (z score) to indicate relative expression across the cell clusters. Left: subsets of lymphoid cells; middle: subsets of CD8$^+$ T cells; right: subsets of CD4$^+$ T cells. **B.** Sunburst chart illustrating the distribution of cell clusters within lymphoid cells.

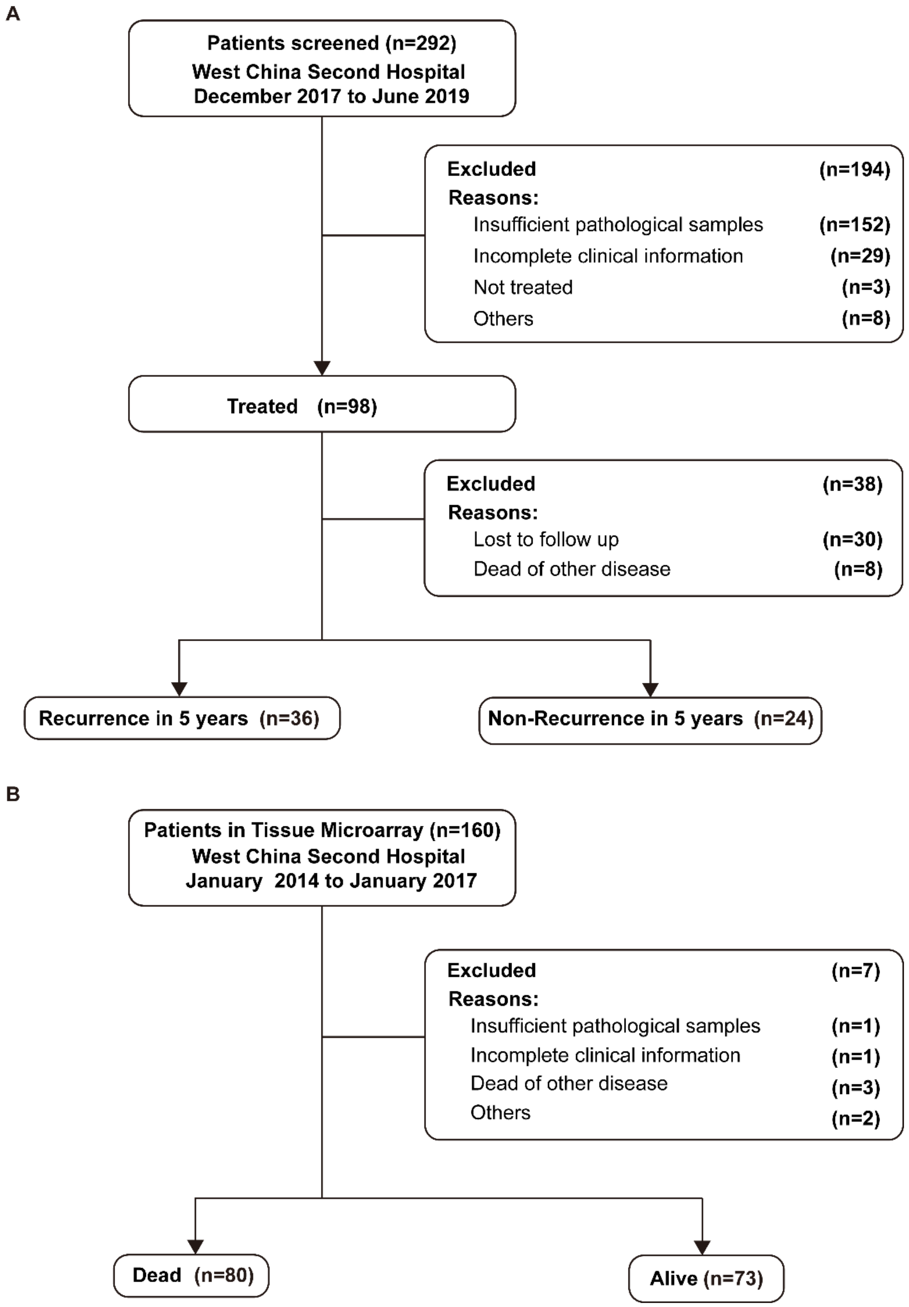

Supplementary Figure 6. Flow diagram of the screening strategy. A. Flow diagram for screening patients who underwent surgical treatment at West China Second Hospital between December 2017 and June 2019. B. Flow diagram for screening patients who underwent surgical treatment at West China Second Hospital between January 2014 and January 2017.

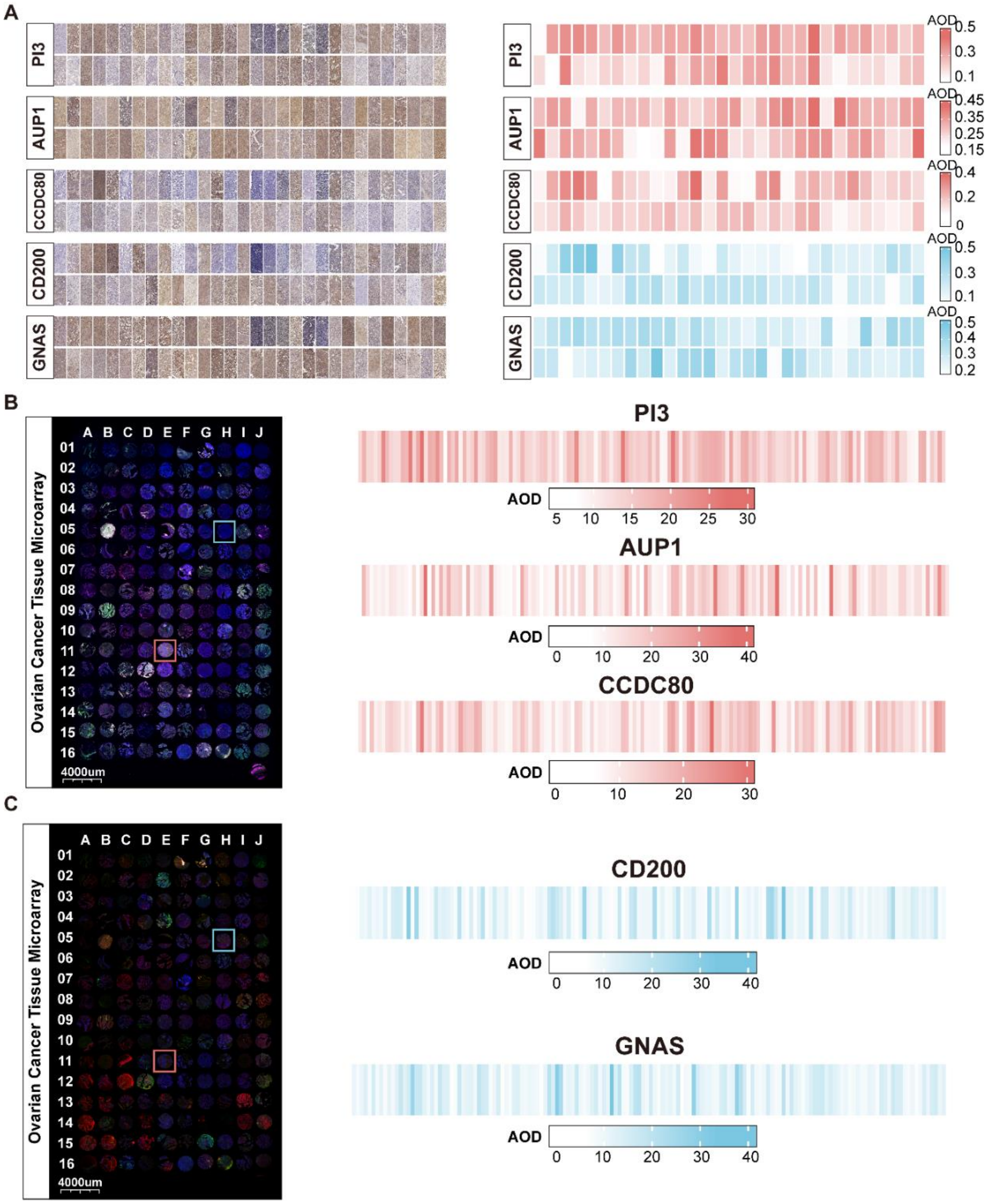

Supplementary Figure 7. Immunohistochemical and immunofluorescence analysis of five key SRGs. A. Representative images (left) and quantification (right) of the relative expression of the PI3, AUP1, CD200, CCDC80, and GNAS proteins via immunohistochemical staining of paraffin-embedded tissue sections from 60 patients. B. Immunofluorescence staining of PI3, AUP1, and CCDC80 in the tissue microarray (n=107) (left). The quantification of the relative expression of PI3, AUP1, and CCDC80 is presented via a heatmap (right). C. Immunofluorescence staining of CD200 and GNAS in the tissue microarray (n=107) (left). The quantification of the relative expression of

CD200 and GNAS is presented via a heatmap (right)

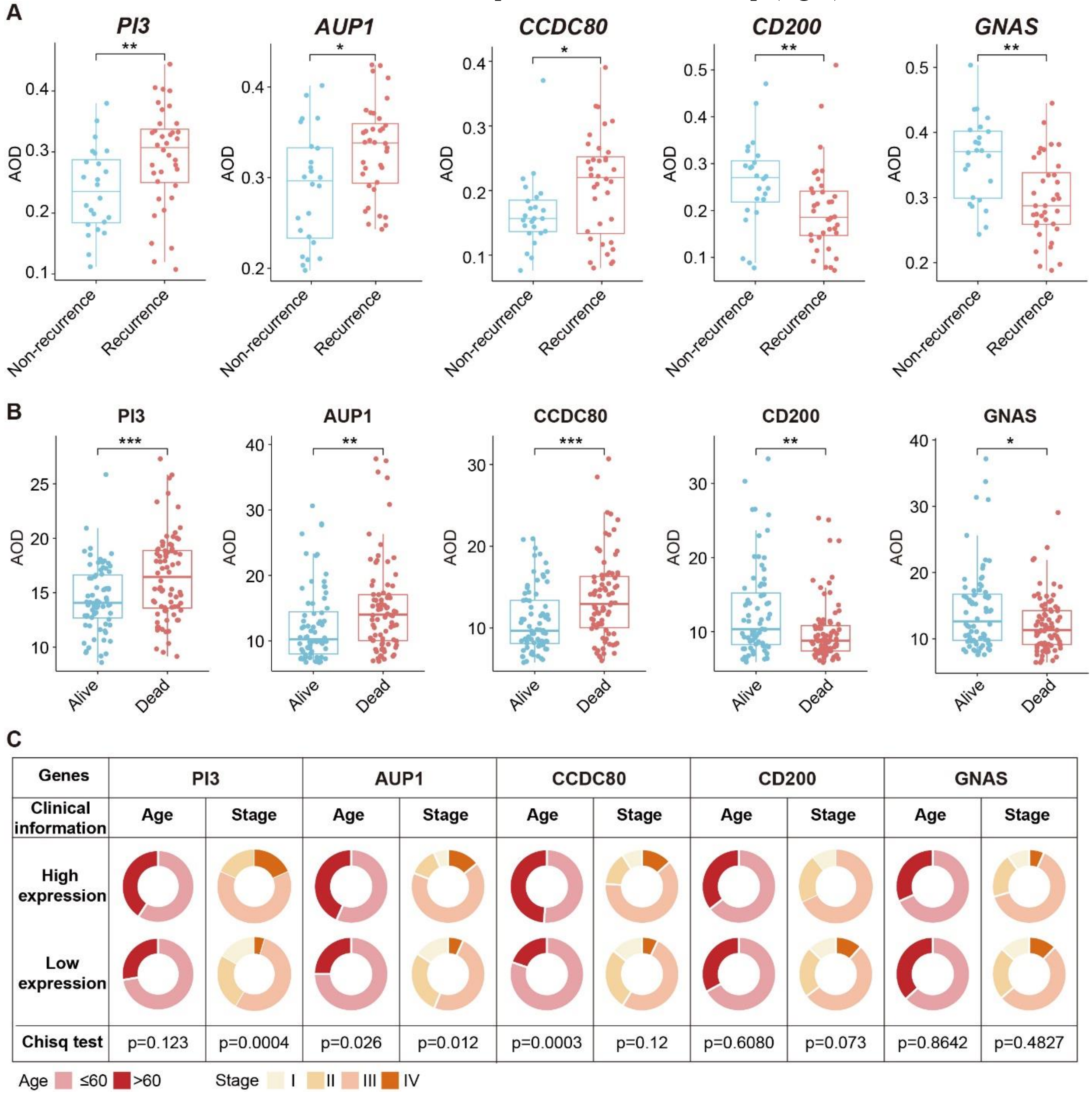

Supplementary Figure 8. Immunohistochemical and immunofluorescence analysis of five key SRGs. A. Boxplots showing differences in the expression of key SEGs (PI3, AUP1, CCDC80, CD200, and GNAS) between recurrent and nonrecurrent patients (n=60). B. Boxplots demonstrating differences in the expression of key SRGs (PI3, AUP1, CCDC80, CD200, and GNAS) between surviving patients and deceased patients (n=153). C. Associations between five SRGs and clinical features.
